# Supplementary figures and images for: NEMF mutations in mice illustrate how Importin-β specific nuclear transport defects recapitulate neurodegenerative disease hallmarks
Source: PLoS Genet. 2024 Sep 23;20(9):e1011411. doi: 10.1371/journal.pgen.1011411 (PMC11449308; doi:10.1371/journal.pgen.1011411)

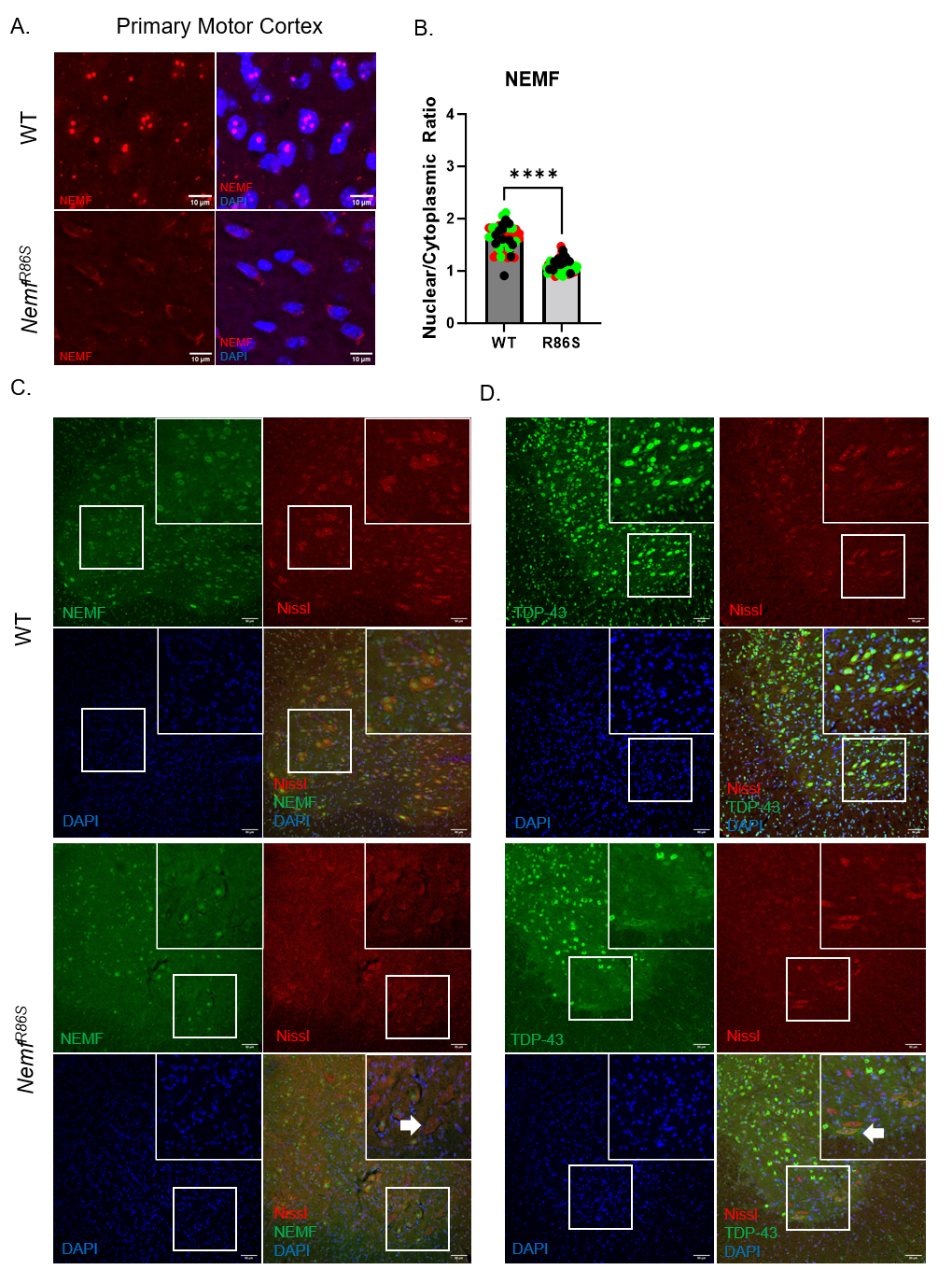

Supplement: S1 Fig — A) Primary Motor Cortex was isolated from 21-day old Wild Type and NemfR86S mice. Neurons in the ventral horn were immunostained for the nucleus (DAPI, blue) and NEMF (red). B) Nuclear/Cytoplasmic ratios of NEMF in WT and NemfR86S. Data analyzed by unpaired two-tailed t-test. C-D) Lumbar Spinal Cords were immunostained for the nucleus (DAPI, blue) and neurons (Nissl, red), and NEMF (C) and TDP-43 (D) (green). Arrows indicate pathological NEMF or TDP-43 Nissl-positive neurons. Individual colors in plots represent one trial. (**** p<0.0001). (TIF) [file pgen.1011411.s001.tif]

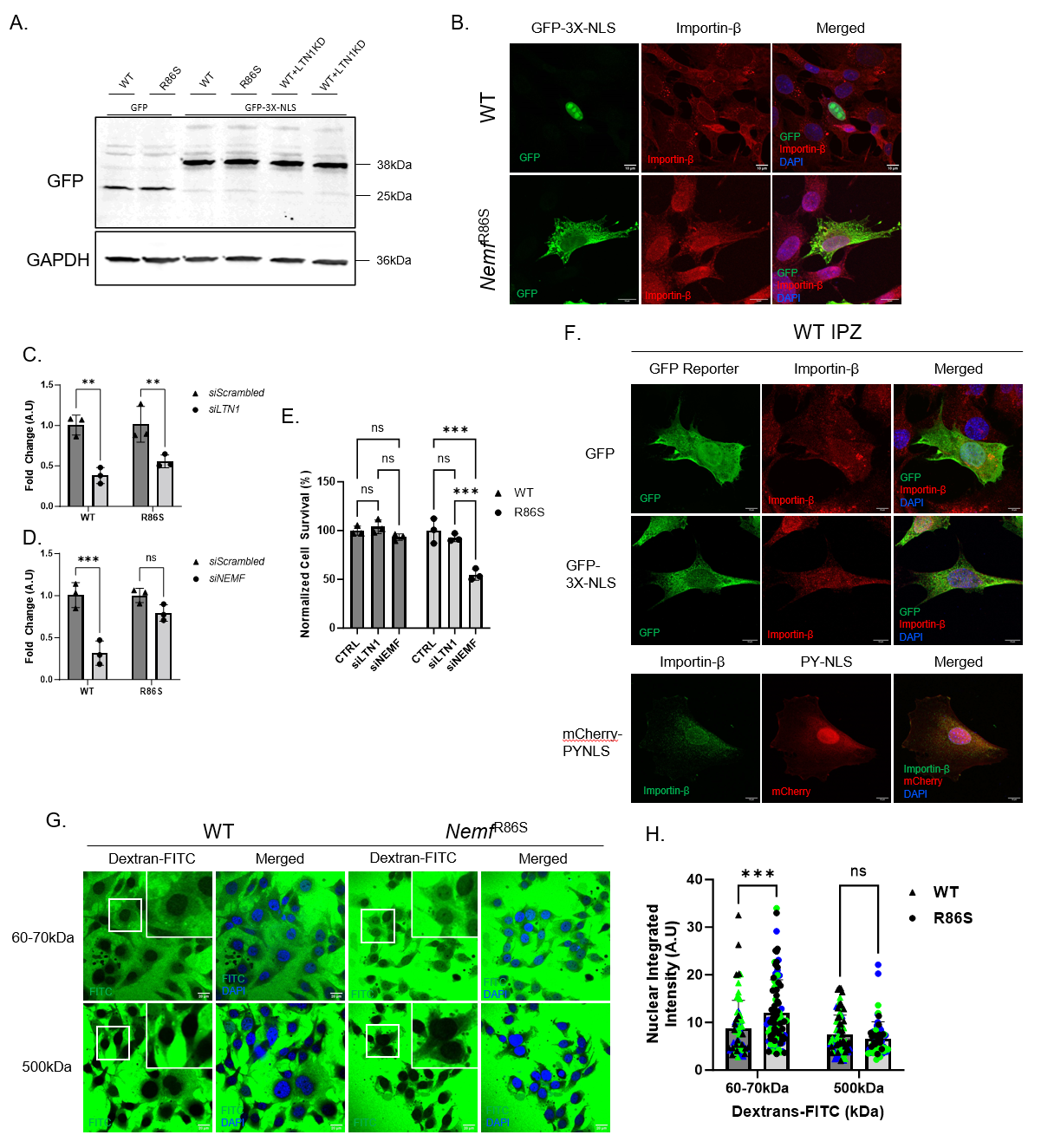

Supplement: S2 Fig — A) Western Blot Analysis of GFP and GFP-3X-NLS expression in WT and NemfR86S MEFs, and in LTN1 and NEMF siRNA treated WT MEFs. B) Expression of GFP-3X-NLS co-stained with Importin-β in WT MEFs. C-D) qPCR validation of Ltn1 and Nemf siRNA knockdown (n = 3). E) Normalized Cell survival of WT and NemfR86S MEFs expressing LTN1 and NEMF siRNA (n = 3). F) Expression of GFP, GFP-3X-NLS, and mCherry-PY-NLS co-stained with Importin-β in WT MEFs treated with IPZ. G) FITC-conjugated dextrans (60-70kDa, and 500kDa). Nuclei labeled with DAPI (blue). H) Quantification of the integrated intensity of dextrans within the nucleus. Individual colors in plots represent one trial. (ns p>0.05, ** p<0.01). (TIF) [file pgen.1011411.s002.tif]

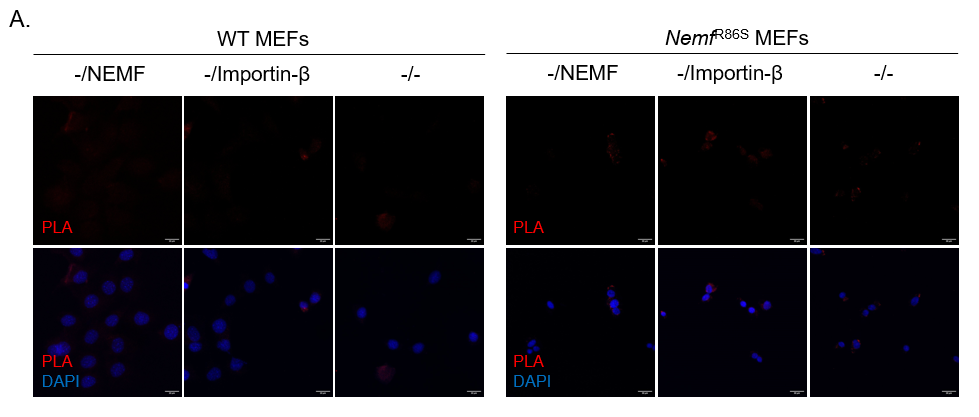

Supplement: S3 Fig — Negative Controls for NEMF and Importin-β PLA A) PLA of NEMF and Importin-β alone, or without primary antibodies (red). Nuclei labeled with DAPI (blue). (TIF) [file pgen.1011411.s003.tif]

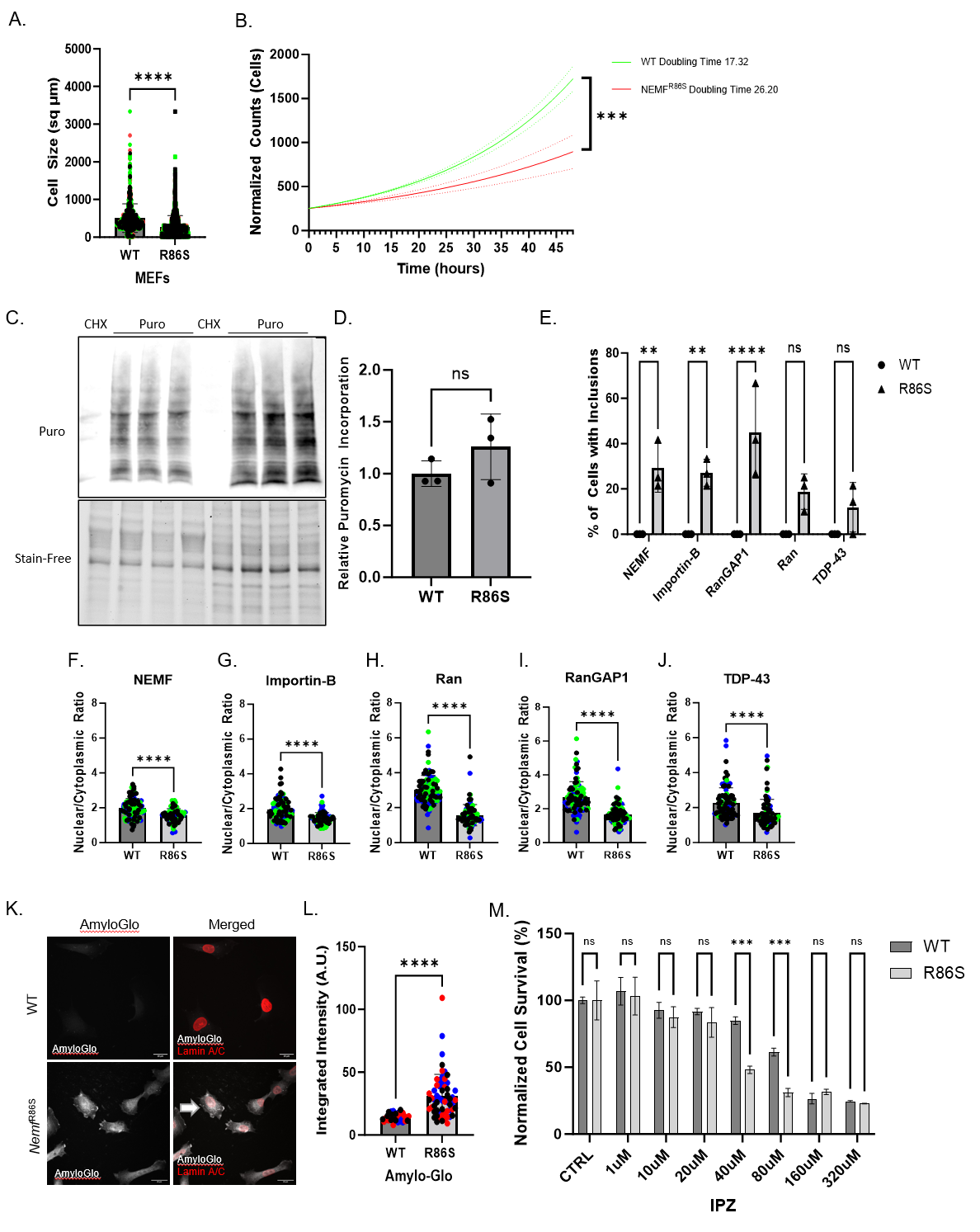

Supplement: S4 Fig — A) Quantification of WT and NemfR86S MEF cell size in square microns using phalloidin as cell maker. Data analyzed by unpaired two-tailed t-test. (n = 999–1000) B) Growth curves of WT (n = 3, r2 = 0.99) and NemfR86S (n = 3, r2 = 0.98) over 48 hours at 15min intervals. Data was analyzed by a nonlinear regression for Malthusian growth. C) Western Blot analysis of global protein synthesis by puromycin immunostaining with or without cycloheximide (CHX) pre-treatment. D) Quantification of puromycin immunostaining standardized to stain-free total protein stain (n = 3). E) Quantification of the percentage of cells with cytoplasmic puncta from Fig 4. Data was analyzed by two-way ANOVA with Šídák’s multiple comparisons test (n = 3). F-J) Quantification of Nuclear/Cytoplasmic Ratio of indicated proteins. Data analyzed by unpaired two-tailed t-test (n = 100). K) Staining of amyloid in WT and NemfR86S MEFs by AmyloGlo. Nuclei labeled with Lamin A/C. L) Quantification of the Integrated Intensity of AmyloGlo. Data analyzed by two-tailed t-test. (n = 30–60) M) Normalized Cell Survival of IPZ treated WT and NemfR86S MEFs (n = 3). Individual colors in plots represent one trial. (ns p>0.05, **p<0.01, ***p<0.0001, ****p<0.0001). (TIF) [file pgen.1011411.s004.tif]

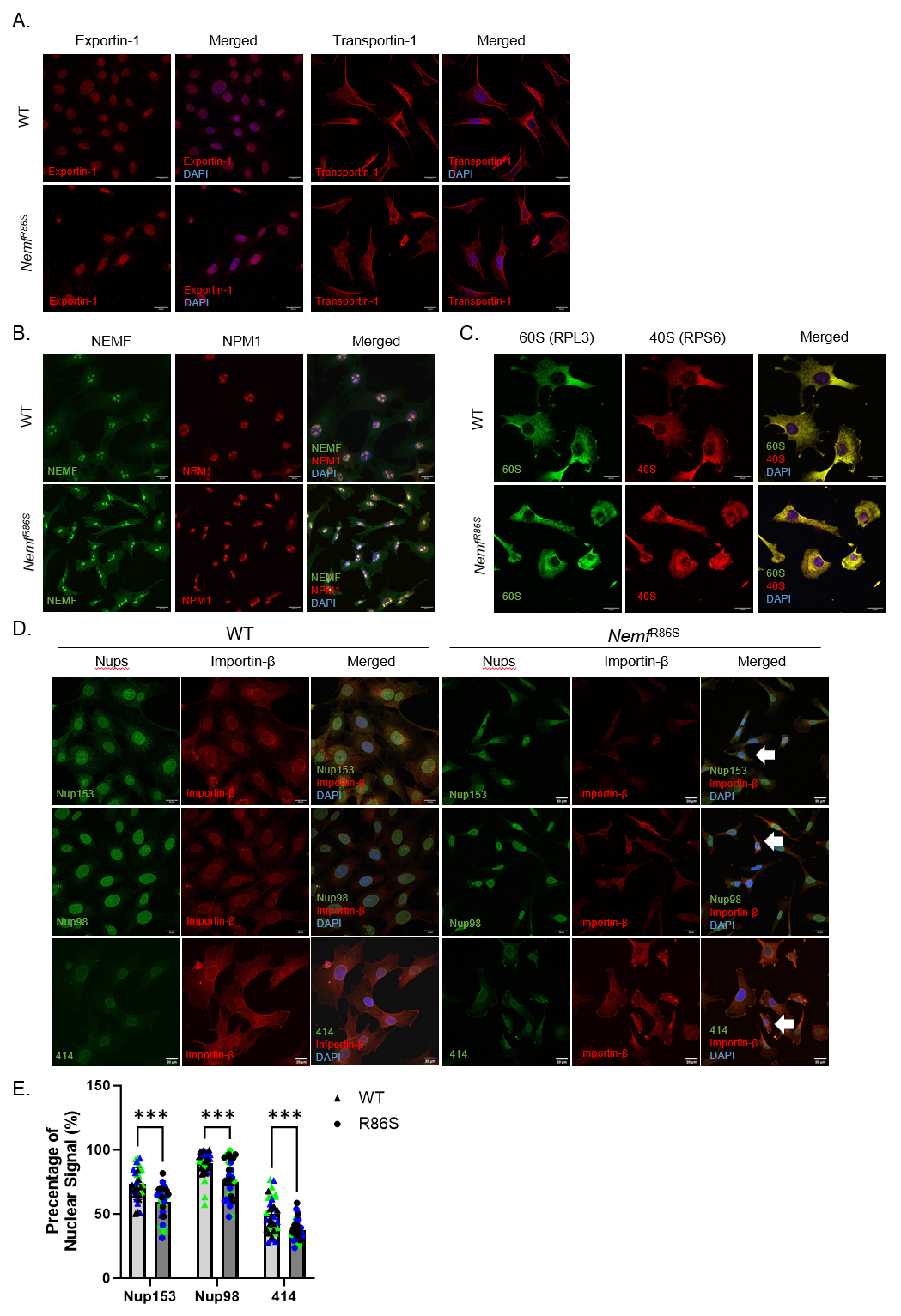

Supplement: S5 Fig — A) Immunostaining of Exportin-1 or Transportin-1 (red) in WT and NemfR86S MEFs. Nuclei labeled with DAPI (blue). B) Immunostaining of NEMF (green) and NPM1 (red) in WT and NemfR86S MEFs. C) Immunostaining of RPL3 (60S, green) and RPS6 (40S, red) in WT and NemfR86S MEFs. D) Immunostaining of Nup153, and Nup98, and mAb414(green) co-stained with Importin-β (red). Nuclei labeled with DAPI (blue). E) Percentage of Nuclear Signal of Nup153, Nup98, and mAb414 in WT and NemfR86S MEFs (n = 30–33 nuclei). Individual colors in plots represent one trial. Scale bars are 20μm. (TIF) [file pgen.1011411.s005.tif]

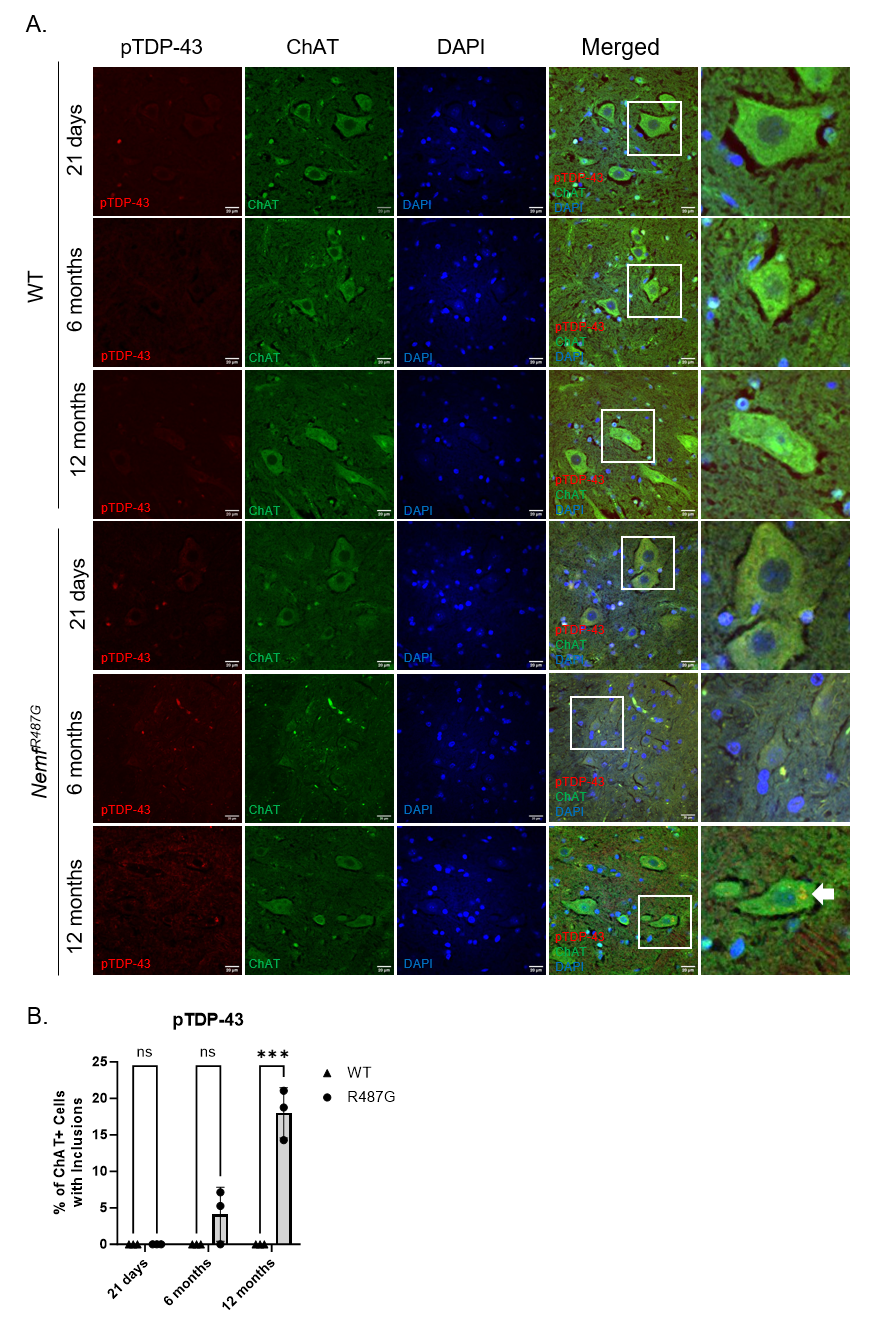

Supplement: S6 Fig — Late onset NemfR487G spinal motor neurons show appearance of phospho-TDP-43 at 6- and 12-months A) Immunofluorescent staining of phospho-TDP-43 (red), ChAT (green) in WT, and NemfR487G lumbar spinal cord motor neurons at 21 days, 6 months, and 12 months. Nuclei labeled with DAPI (blue). B) Percentage of ChAT+ cells with pTDP-43 cytoplasmic inclusions in WT and NemfR487G mice (n = 3). Data analyzed by two-way anova with Šídák’s multiple comparisons test. (n = 3). (ns p>0.05, *** p<0.001). (TIF) [file pgen.1011411.s006.tif]

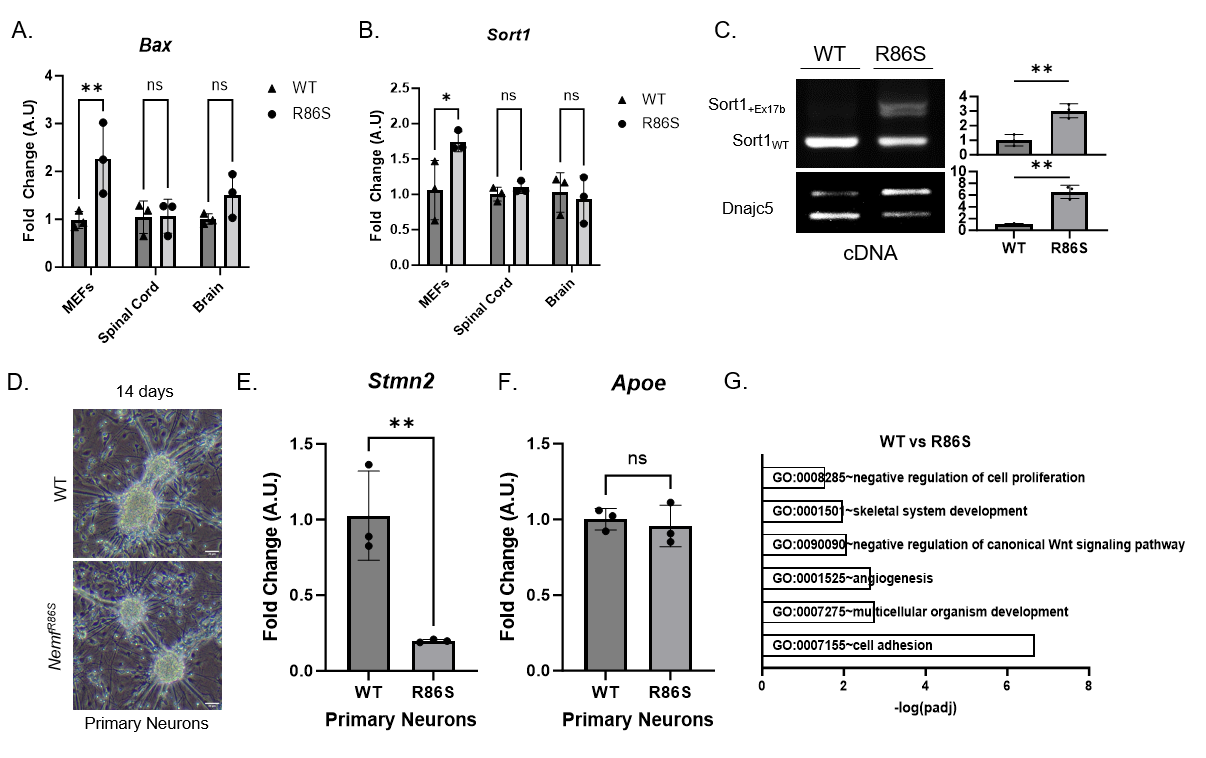

Supplement: S7 Fig — A-B) qPCR relative fold change of Bax and Sorl1 in MEFs, spinal cord, brain in WT and NemfR86S mice. Data analyzed by two-way anova with Šídák’s multiple comparisons test. (n = 3). C) Relative expression of Sort1 and Dnajc5 exon splicing inclusion from WT and NemfR86S MEFs cDNA. D) Brightfield Images of WT and NemfR86S Primary Neurons at 14 days post-plating. E-F) qPCR relative fold change of Stmn2 and Apoe in WT and NemfR86S primary neurons (n = 3). G) Gene Ontology Analysis of significantly dysregulated genes in NemfR86S (ns p>0.05, *p<0.05, **p<0.01). (TIF) [file pgen.1011411.s007.tif]

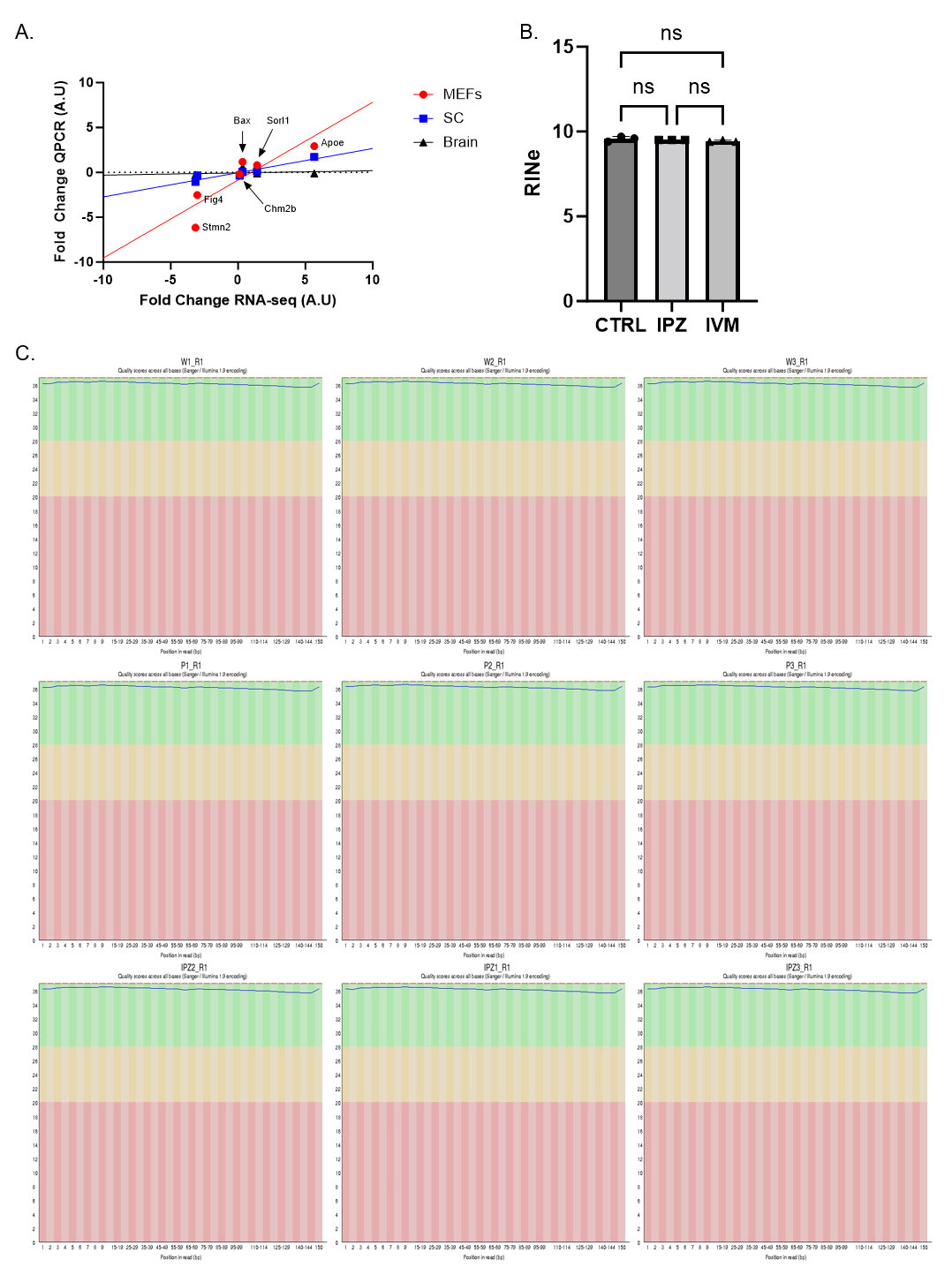

Supplement: S8 Fig — A) qPCR log2FoldChange plotted over RNA for MEFs (r2 = .76,*p<0.05), Spinal Cord (SC) (r2 = .89,**p<0.01), and Brain (r2 = .05,*p>0.05). A simple linear regression determined the best-fit model. B) Tapestation Analysis comparing RNA integrity number for WT, IPZ-treated, and IVM-treated RNA samples. Data analyzed by one-way anova with Tukey’s multiple comparison test. C) RNA quality scores throughout Sanger Sequencing Position as presented by Azenta. (ns p>0.05). (TIF) [file pgen.1011411.s008.tif]

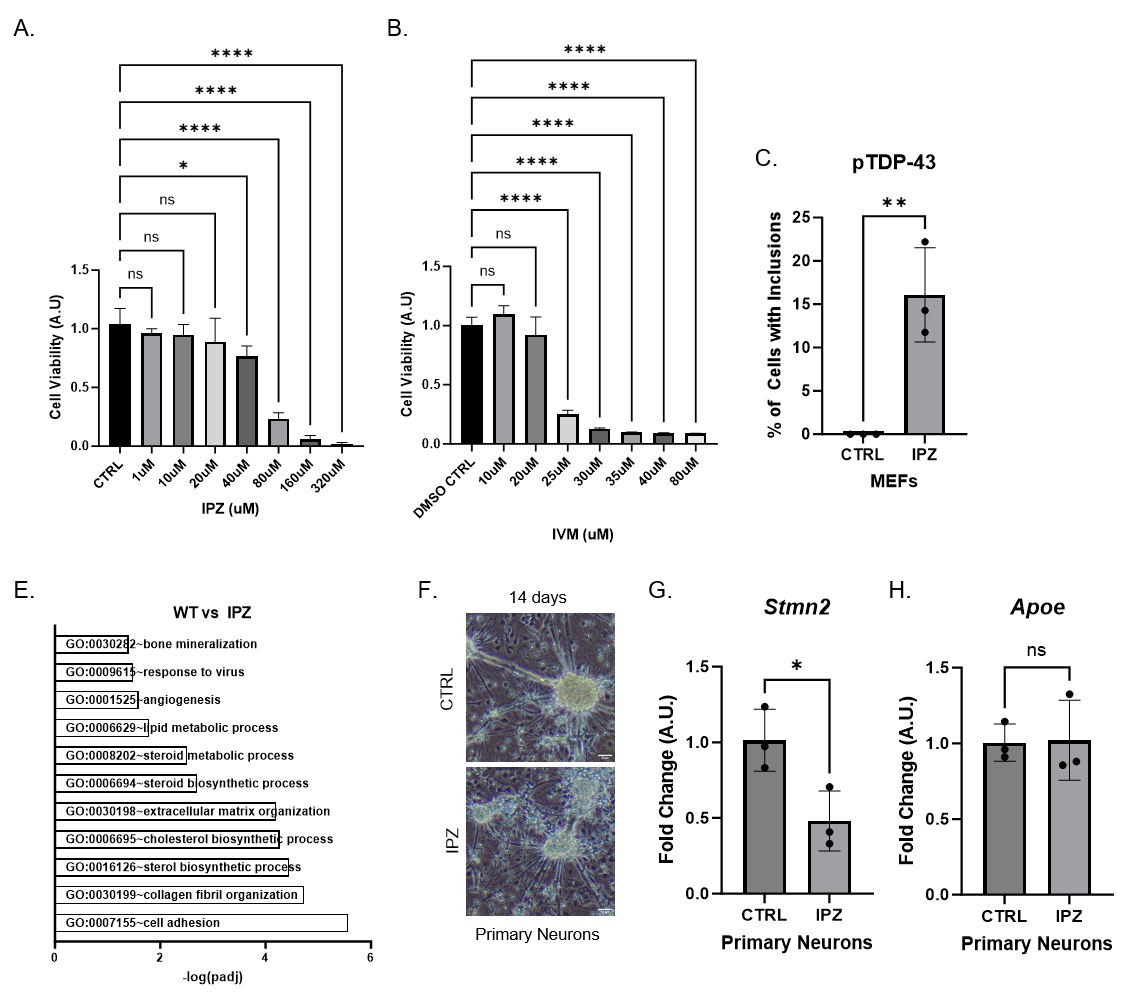

Supplement: S9 Fig — IPZ and IVM Cell Viability Curves and Gene Ontology Analyses A-B) Cell Viability Curves for IPZ (A) and IVM (B) treated MEFs. Data analyzed by one-way with Tukey’s multiple comparison test C) Percentage of cells with pTDP-43 cytoplasmic inclusions in DMSO-CTRL and IPZ-treated WT MEFs (n = 3). E) Gene Ontology Analysis of significantly dysregulated genes in NemfR86S and IPZ-treated samples. F) Brightfield Images of DMSO-CTRL and IPZ-treated WT Primary Neurons at 14 days post-plating. G-H) qPCR relative fold change of Stmn2 and Apoe in WT and IPZ-treated primary neurons (n = 3). (ns p>0.05, *p<0.05, **p<0.01, **** p<0.0001). (TIF) [file pgen.1011411.s009.tif]

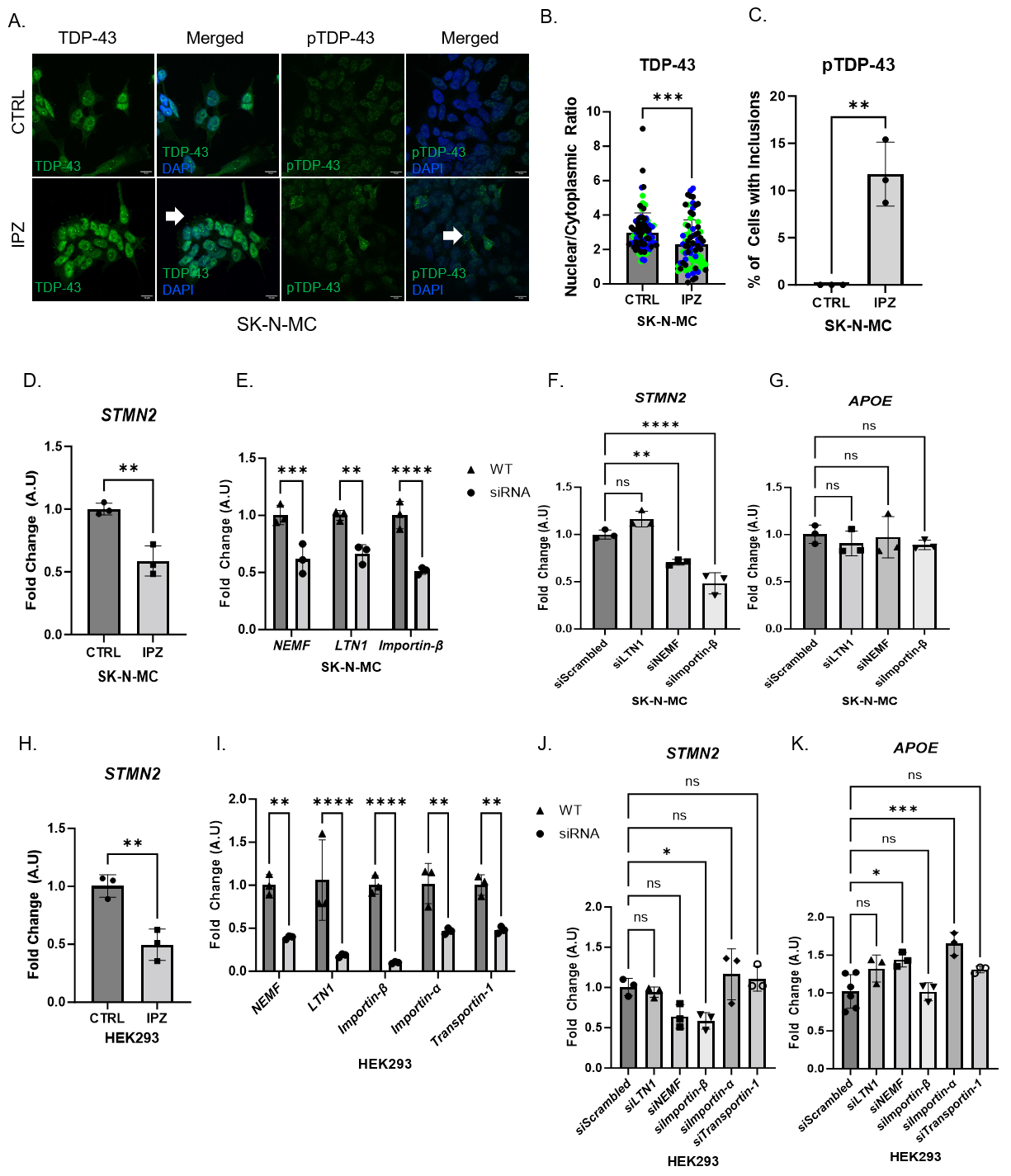

Supplement: S10 Fig — qPCR Validation and STMN2 expression in HEK293 cells A) Immunofluorescent staining of TDP-43 and pTDP-43 in DMSO control and Importazole-treated (IPZ) SK-N-MC (neuroblastoma) cells. B) Quantification of Nuclear/Cytoplasmic Ratio of TDP-43 (n = 80–96). C) Percentage of cells with pTDP-43 cytoplasmic inclusions in DMSO contorl and IPZ-treated (n = 3). D) qPCR relative fold change in DMSO control and IPZ-treated SK-N-MC cells for STMN2 (n = 3). E) qPCR validation of NEMF, LTN1, and Importin-β siRNA knockdown in SK-N-MC cells (n = 3). F-G) qPCR relative fold change of STMN2 or APOE treated with LTN1, NEMF, or Importin-β siRNAs in SK-N-MC cells (n = 3). Data analyzed by one-way ANOVA with Tukey’s multiple comparison test. H) qPCR relative fold change in DMSO Control and Importazole (IPZ) treated HEK293 for STMN2 (n = 3). I) qPCR validation of LTN1 (RNF160), NEMF (SDCCAG1), Importin-β (KPNB), Importin-α (KPNA), or Transportin-1 (TPNO1) siRNA knockdown in HEK293 cells (n = 3). Data analyzed by two-way ANOVA with Šídák’s multiple comparisons test. J-K) qPCR relative fold change of STMN2 or APOE treated with LTN1, NEMF, Importin-β, Importin-α, or Transportin-1siRNAs in HEK293 cells (n = 3). Data analyzed by one-way anova with Tukey’s multiple comparison test. Individual colors in plots represent one trial. (ns p>0.05, *p<0.05, **p<0.01, ***p<0.001 **** p<0.0001). (TIF) [file pgen.1011411.s010.tif]

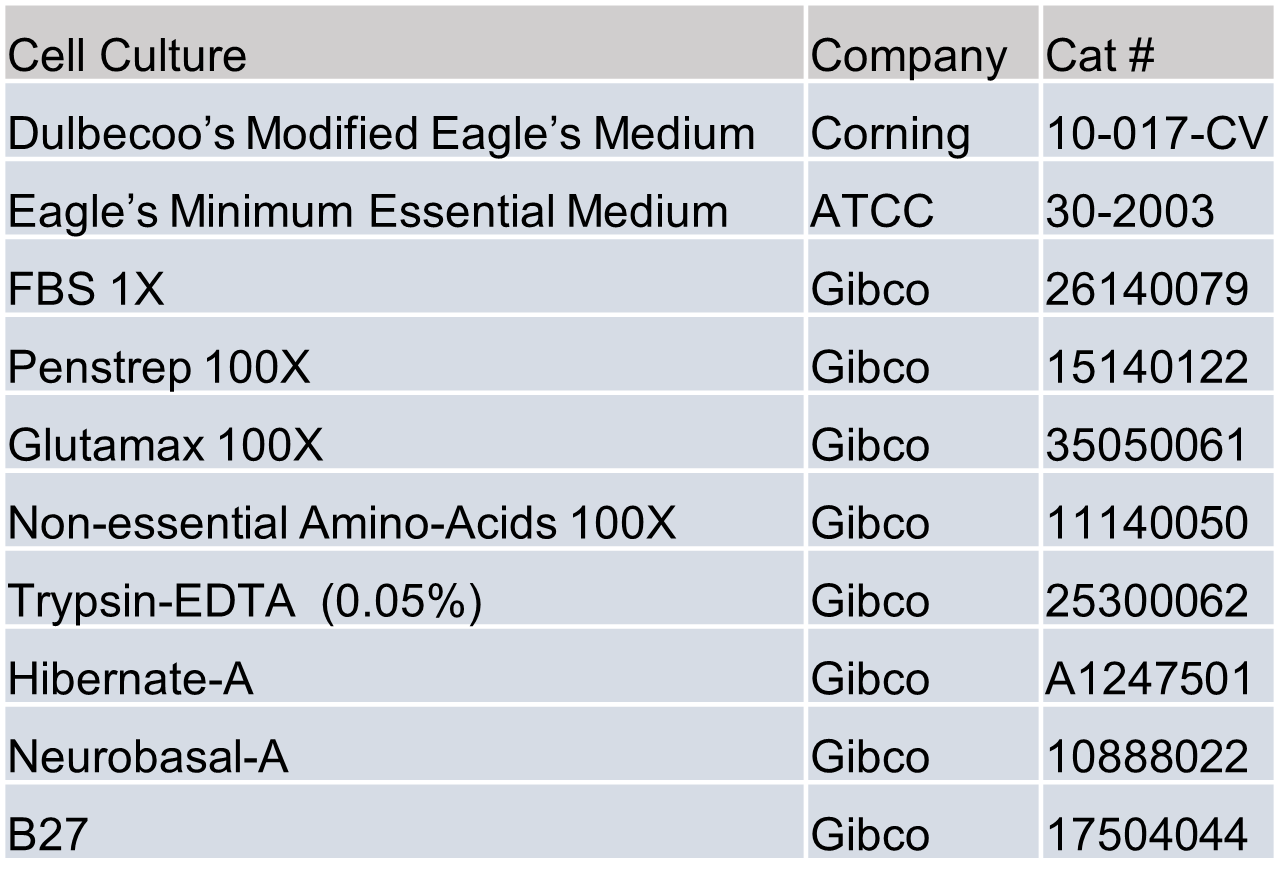

Supplement: S1 Table — (TIF) [file pgen.1011411.s011.tif]

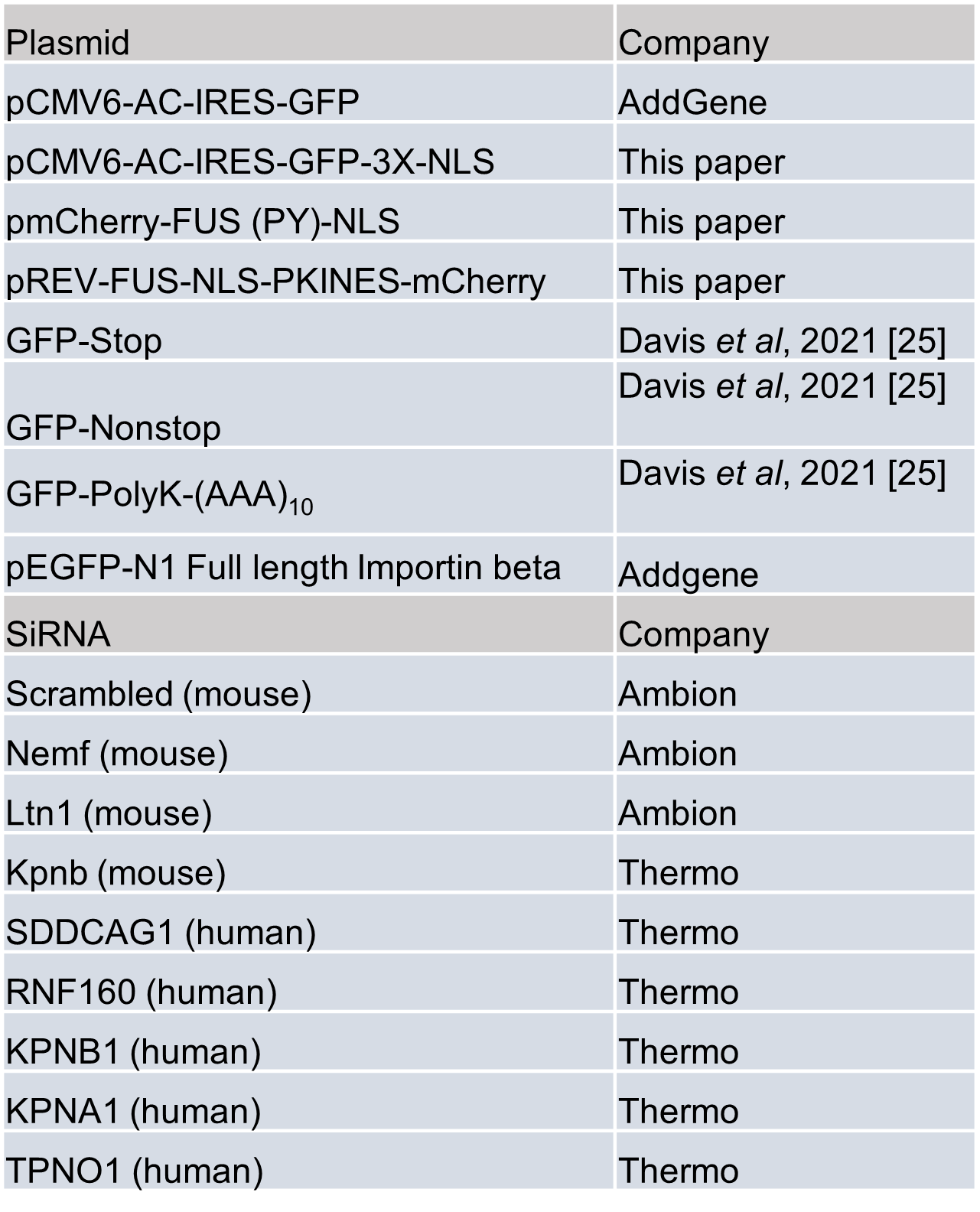

Supplement: S2 Table — GFP-Stop, GFP-Nonstop, and GFP-PolyK-(AAA)10 plasmids were previously described in Davis et al, 2021 [25]. (TIF) [file pgen.1011411.s012.tif]

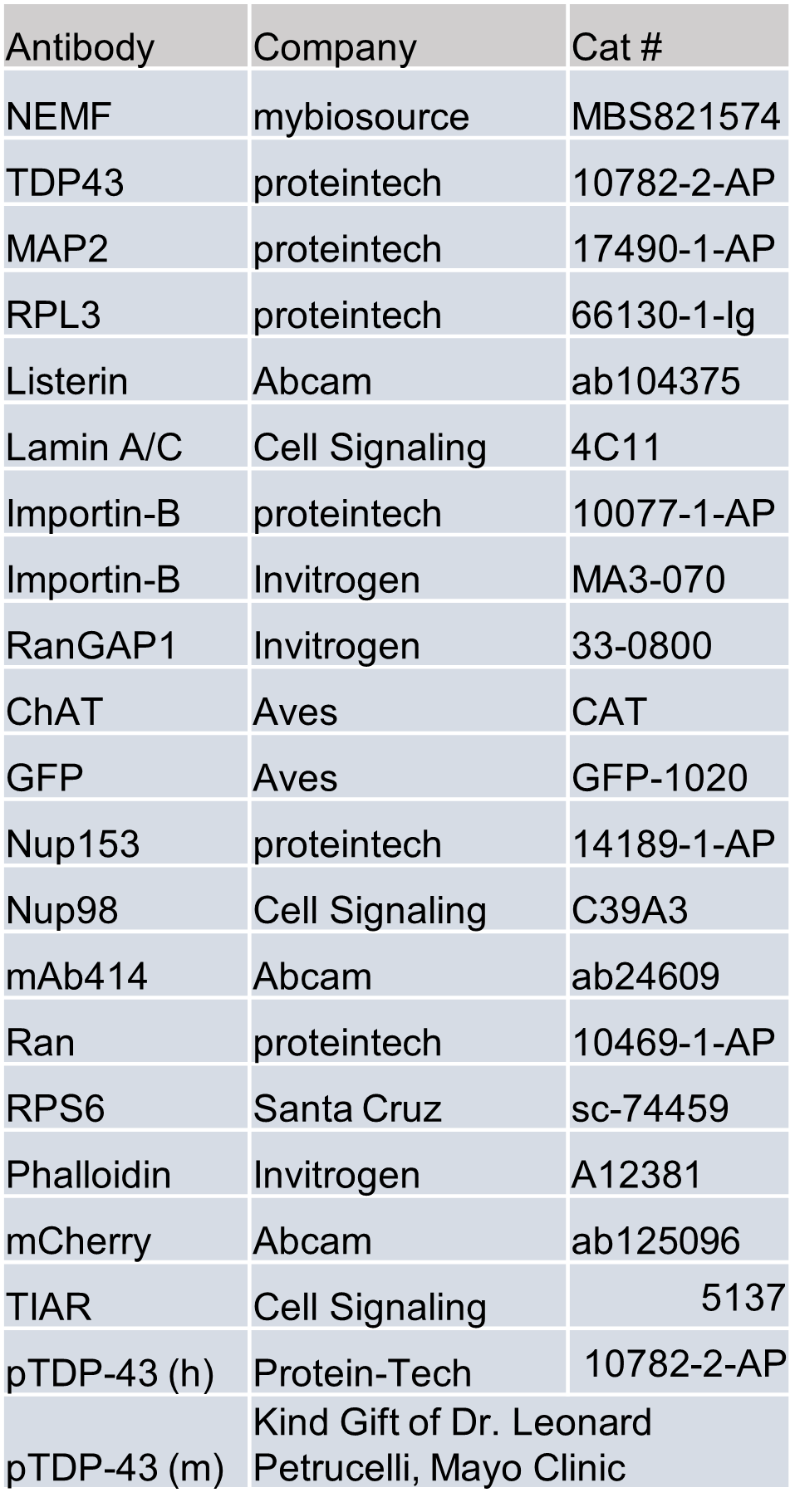

Supplement: S3 Table — (TIF) [file pgen.1011411.s013.tif]

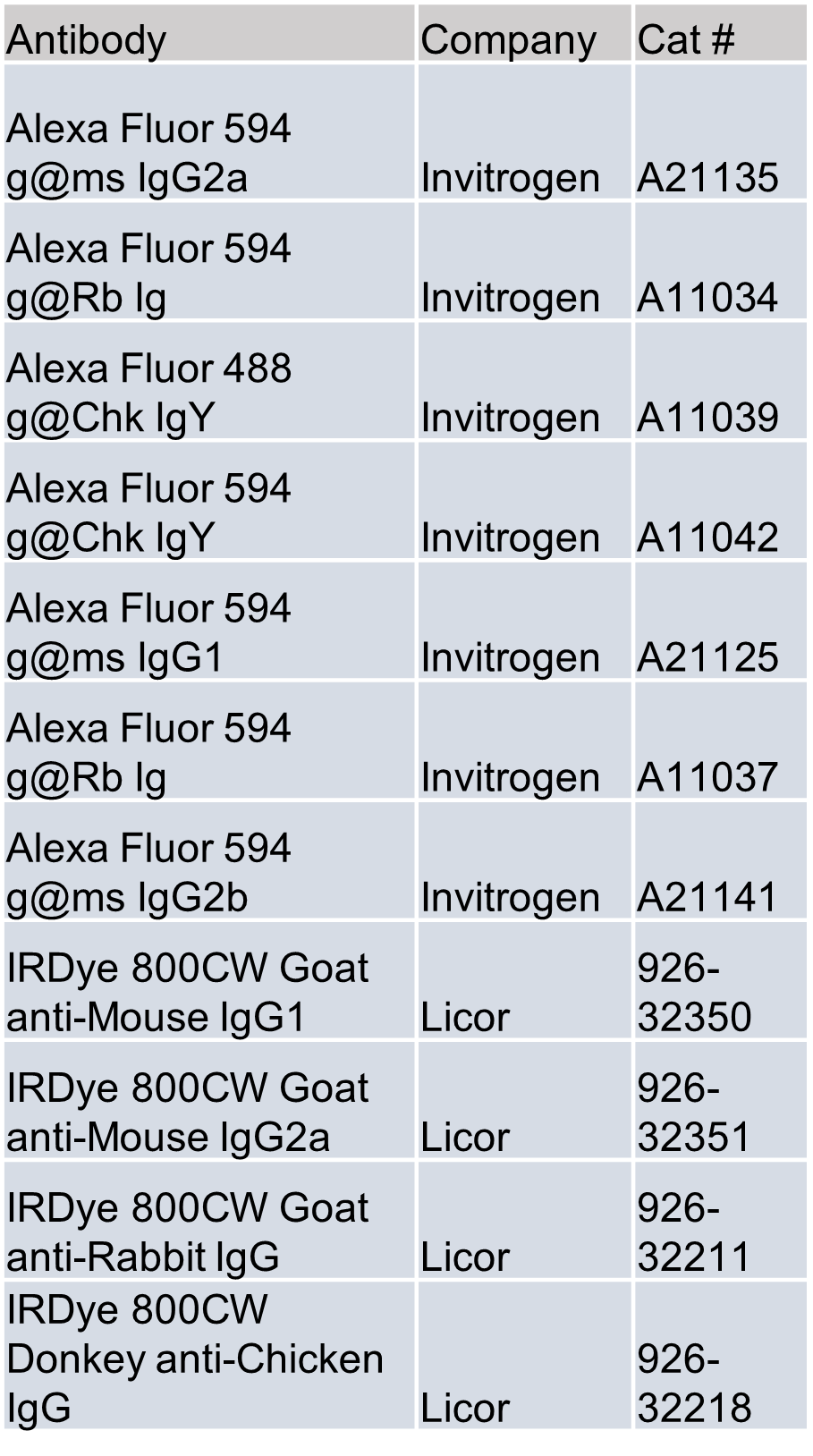

Supplement: S4 Table — (TIF) [file pgen.1011411.s014.tif]

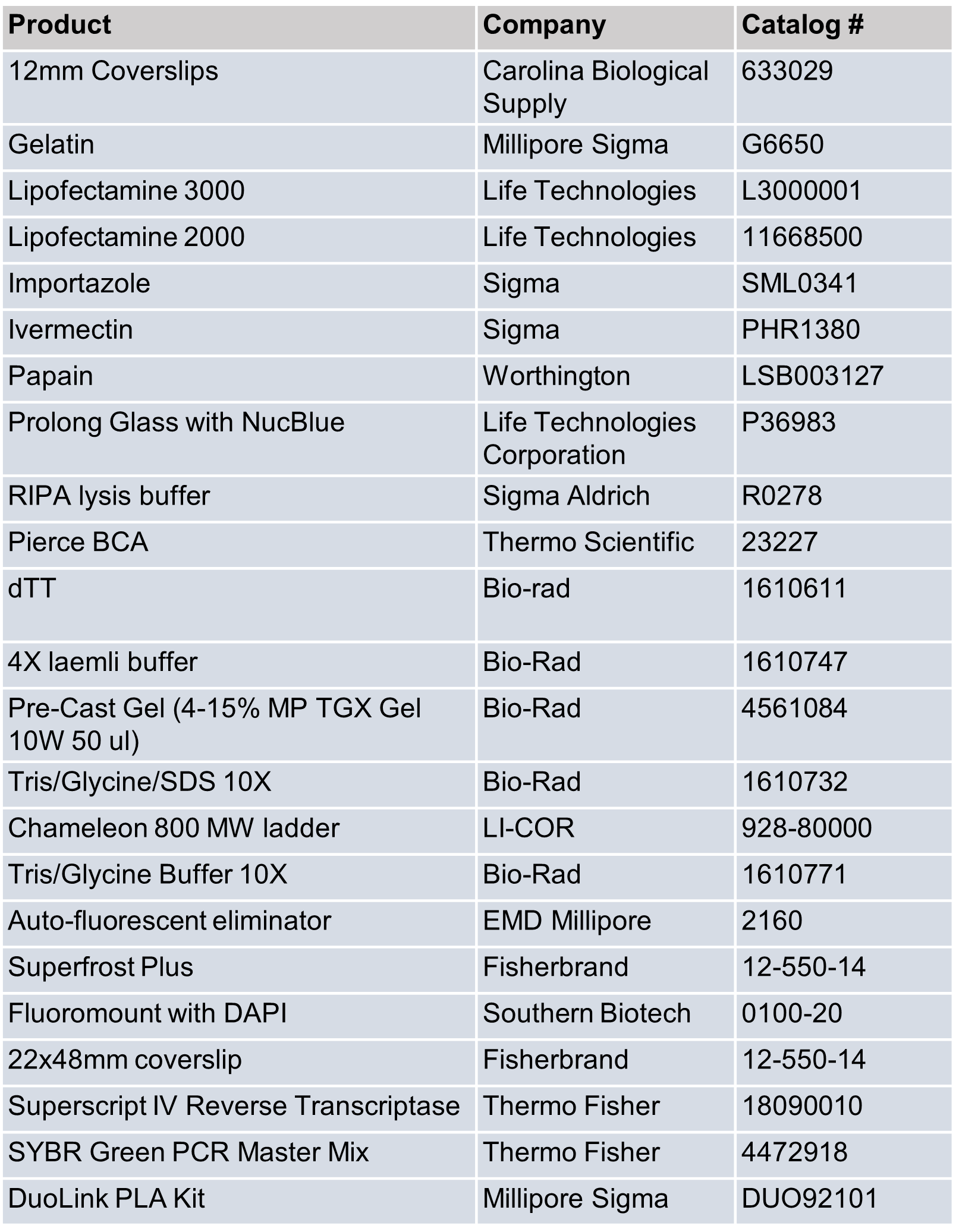

Supplement: S5 Table — (TIF) [file pgen.1011411.s015.tif]

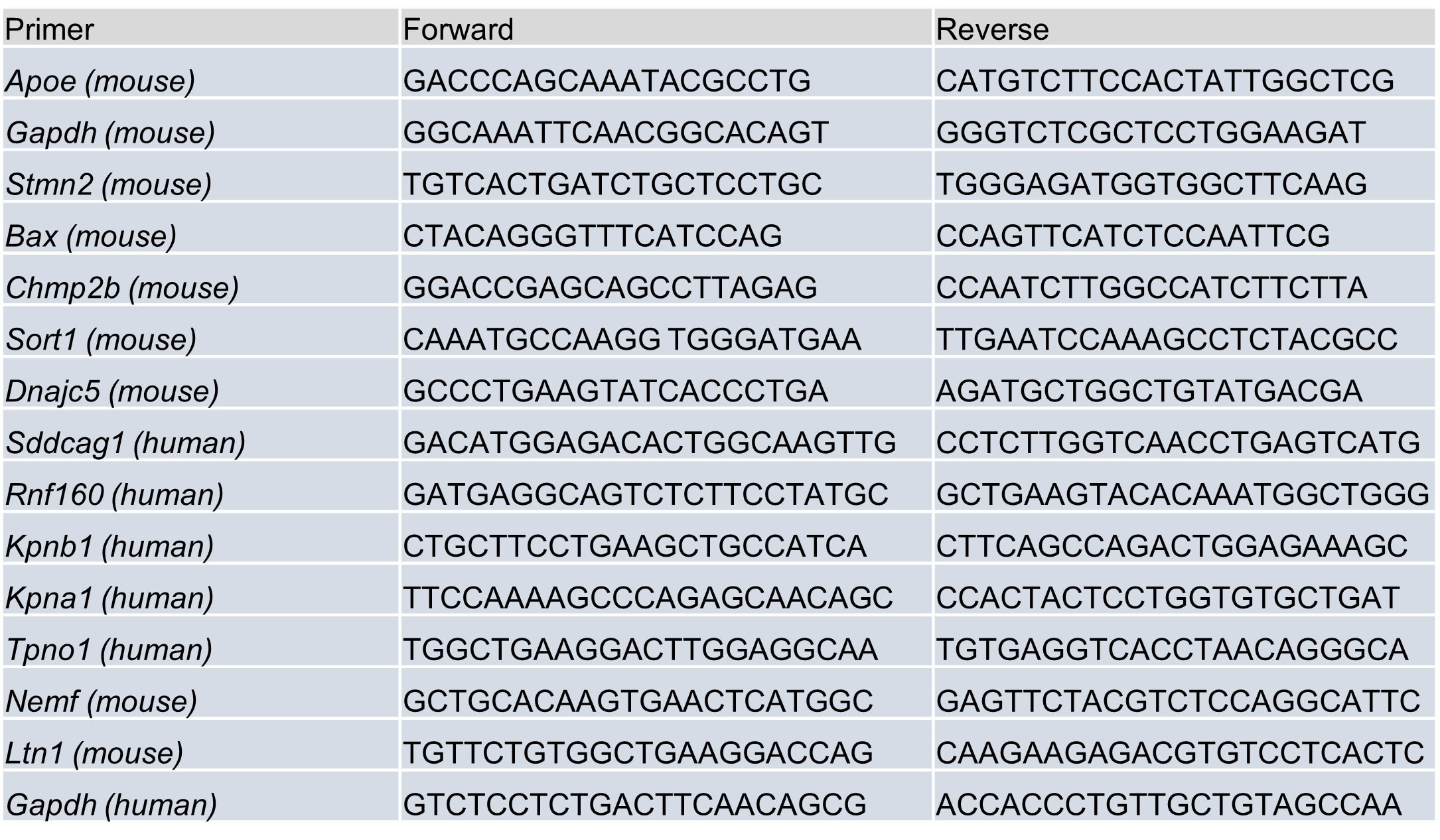

Supplement: S6 Table — (TIF) [file pgen.1011411.s016.tif]

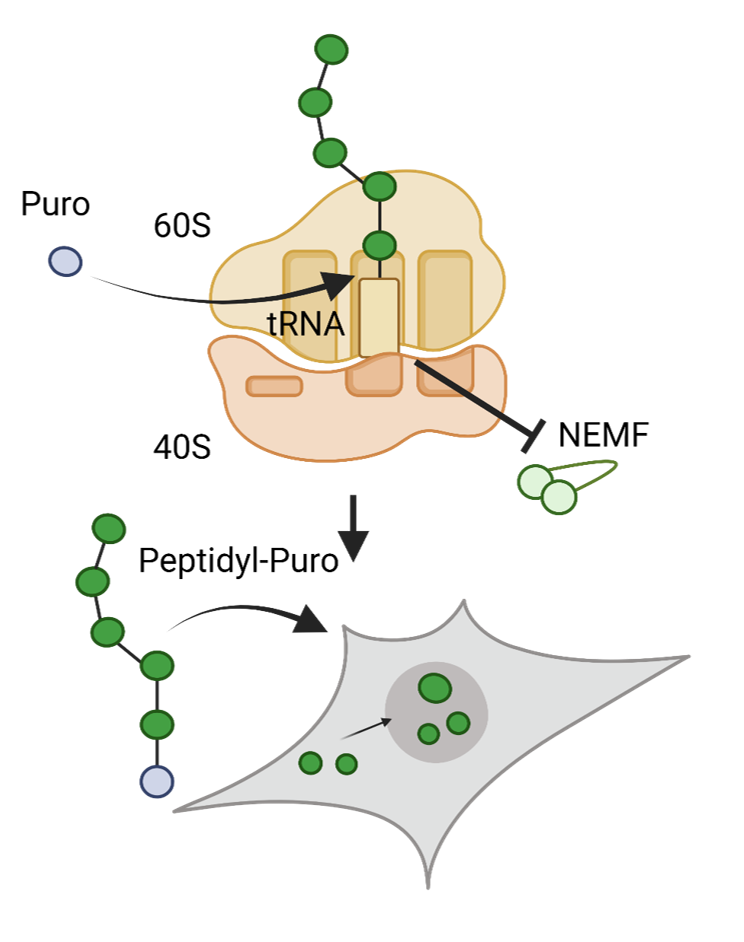

Supplement: S1 Data — (ZIP) [file pgen.1011411.s017.zip › S17 Data/Fig3/Biorender_image_2.png]

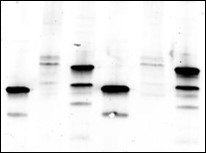

Supplement: S1 Data — (ZIP) [file pgen.1011411.s017.zip › S17 Data/Fig3/GFP_reporter_blot.jpg]

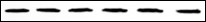

Supplement: S1 Data — (ZIP) [file pgen.1011411.s017.zip › S17 Data/Fig3/GFP_reporter_controls.jpg]

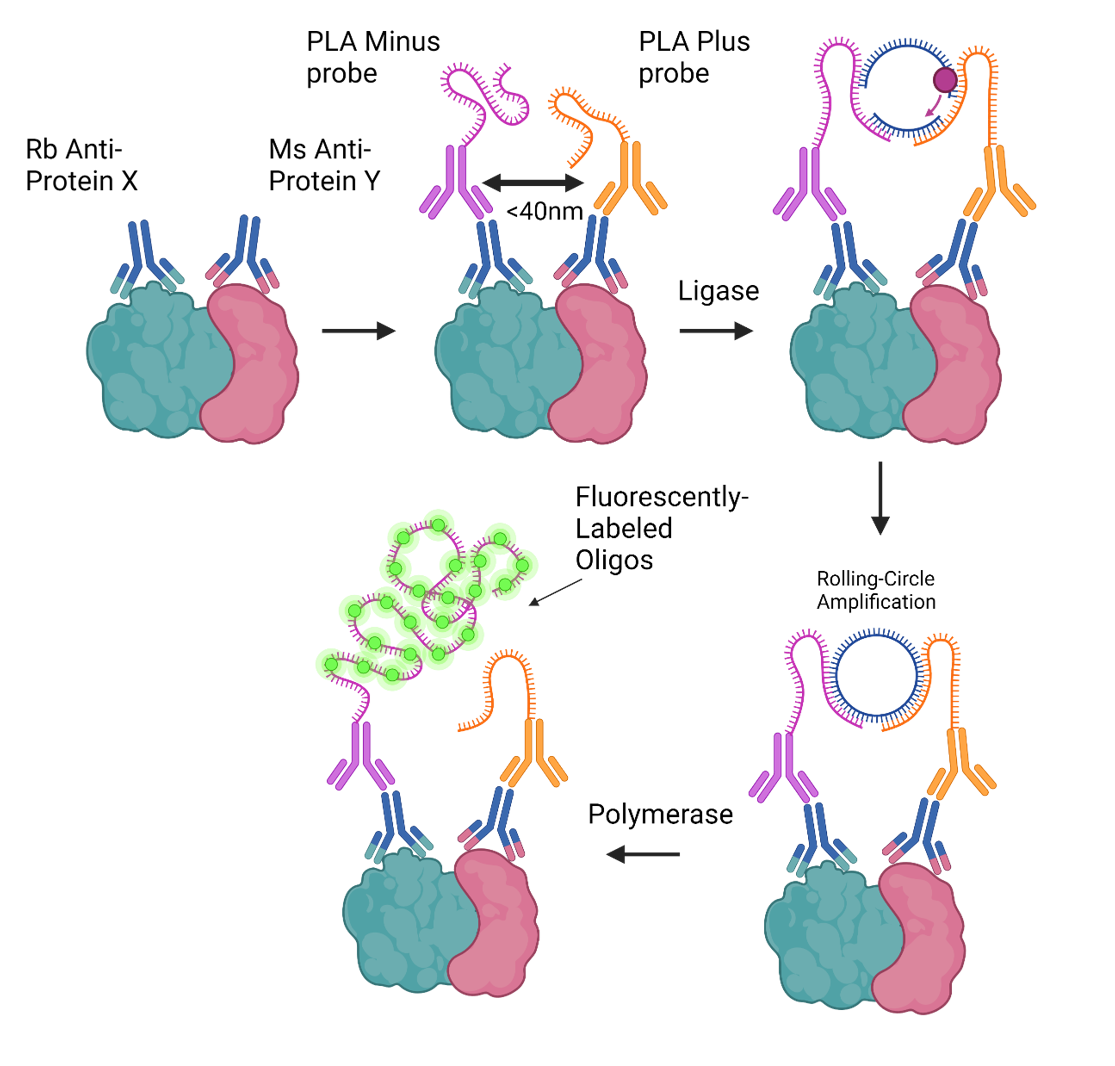

Supplement: S1 Data — (ZIP) [file pgen.1011411.s017.zip › S17 Data/Fig4/Biorender_image_1.png]

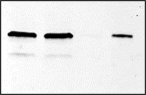

Supplement: S1 Data — (ZIP) [file pgen.1011411.s017.zip › S17 Data/Fig5/TDP_43_Ins_blot.png]

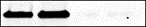

Supplement: S1 Data — (ZIP) [file pgen.1011411.s017.zip › S17 Data/Fig5/TDP_43_Ins_gapdh_blot.png]

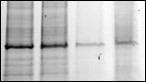

Supplement: S1 Data — (ZIP) [file pgen.1011411.s017.zip › S17 Data/Fig5/TDP_43_Ins_stain-free.jpg]

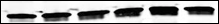

Supplement: S1 Data — (ZIP) [file pgen.1011411.s017.zip › S17 Data/Fig6/gapdh_brain.png]

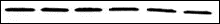

Supplement: S1 Data — (ZIP) [file pgen.1011411.s017.zip › S17 Data/Fig6/Gapdh_mefs.png]

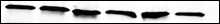

Supplement: S1 Data — (ZIP) [file pgen.1011411.s017.zip › S17 Data/Fig6/Gapdh_SC.png]

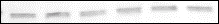

Supplement: S1 Data — (ZIP) [file pgen.1011411.s017.zip › S17 Data/Fig6/Stmn2_brain.png]

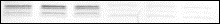

Supplement: S1 Data — (ZIP) [file pgen.1011411.s017.zip › S17 Data/Fig6/Stmn2_mefs.png]

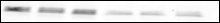

Supplement: S1 Data — (ZIP) [file pgen.1011411.s017.zip › S17 Data/Fig6/Stmn2_SC.png]

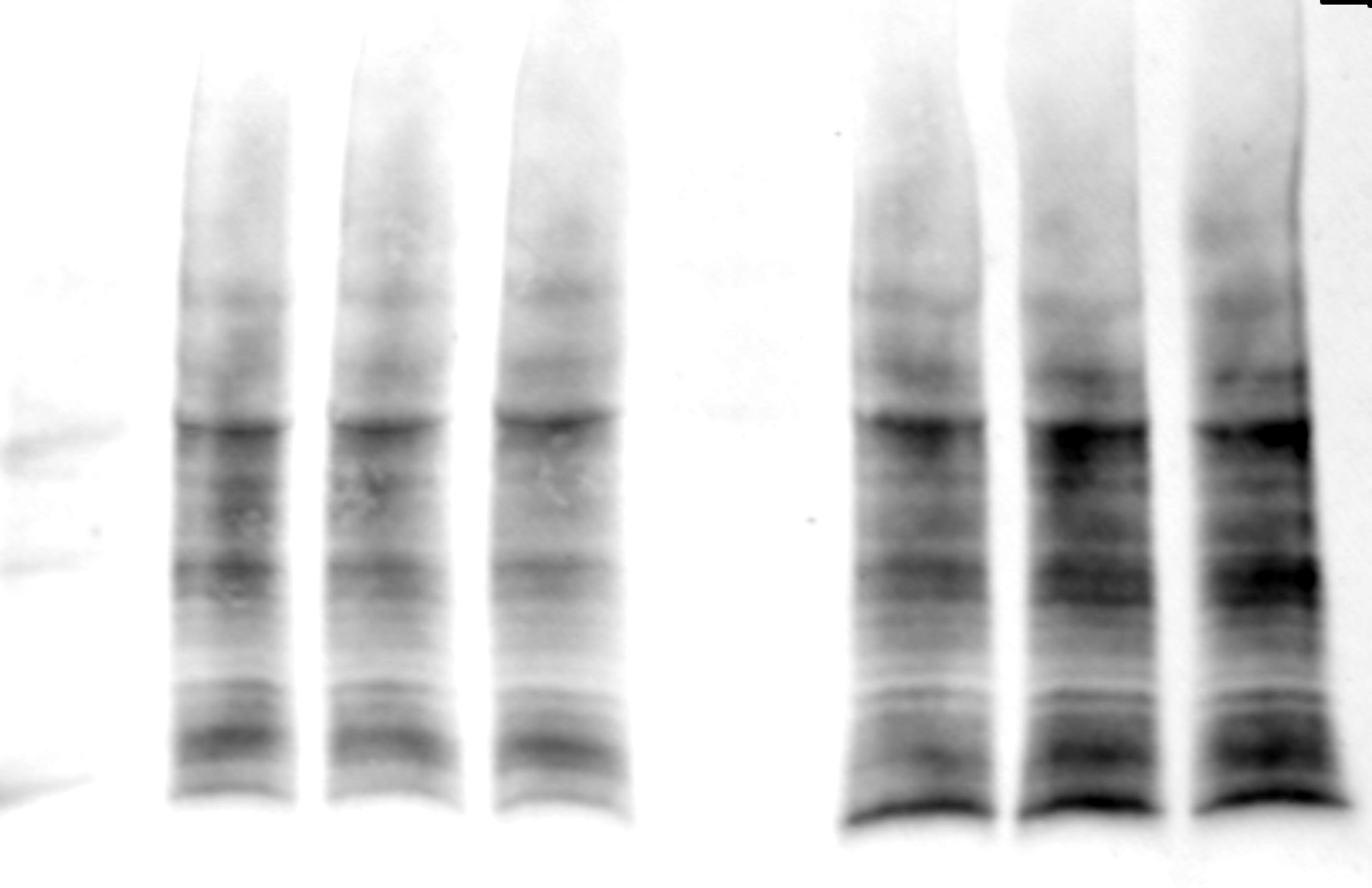

Supplement: S1 Data — (ZIP) [file pgen.1011411.s017.zip › S17 Data/FigS4/MEFS_Puro.jpg]

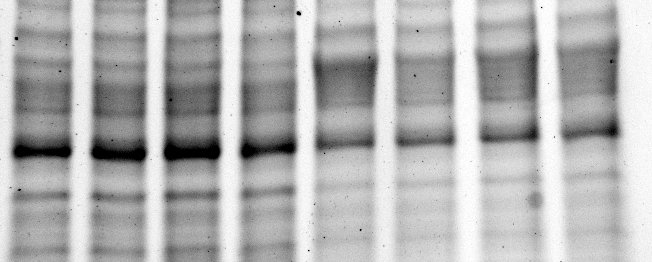

Supplement: S1 Data — (ZIP) [file pgen.1011411.s017.zip › S17 Data/FigS4/Puromycin_assay.jpg]

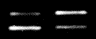

Supplement: S1 Data — (ZIP) [file pgen.1011411.s017.zip › S17 Data/FigS7/Dnajc5_cDNA_Gel.png]

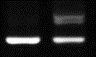

Supplement: S1 Data — (ZIP) [file pgen.1011411.s017.zip › S17 Data/FigS7/Sort1_cDNA_Gel.png]

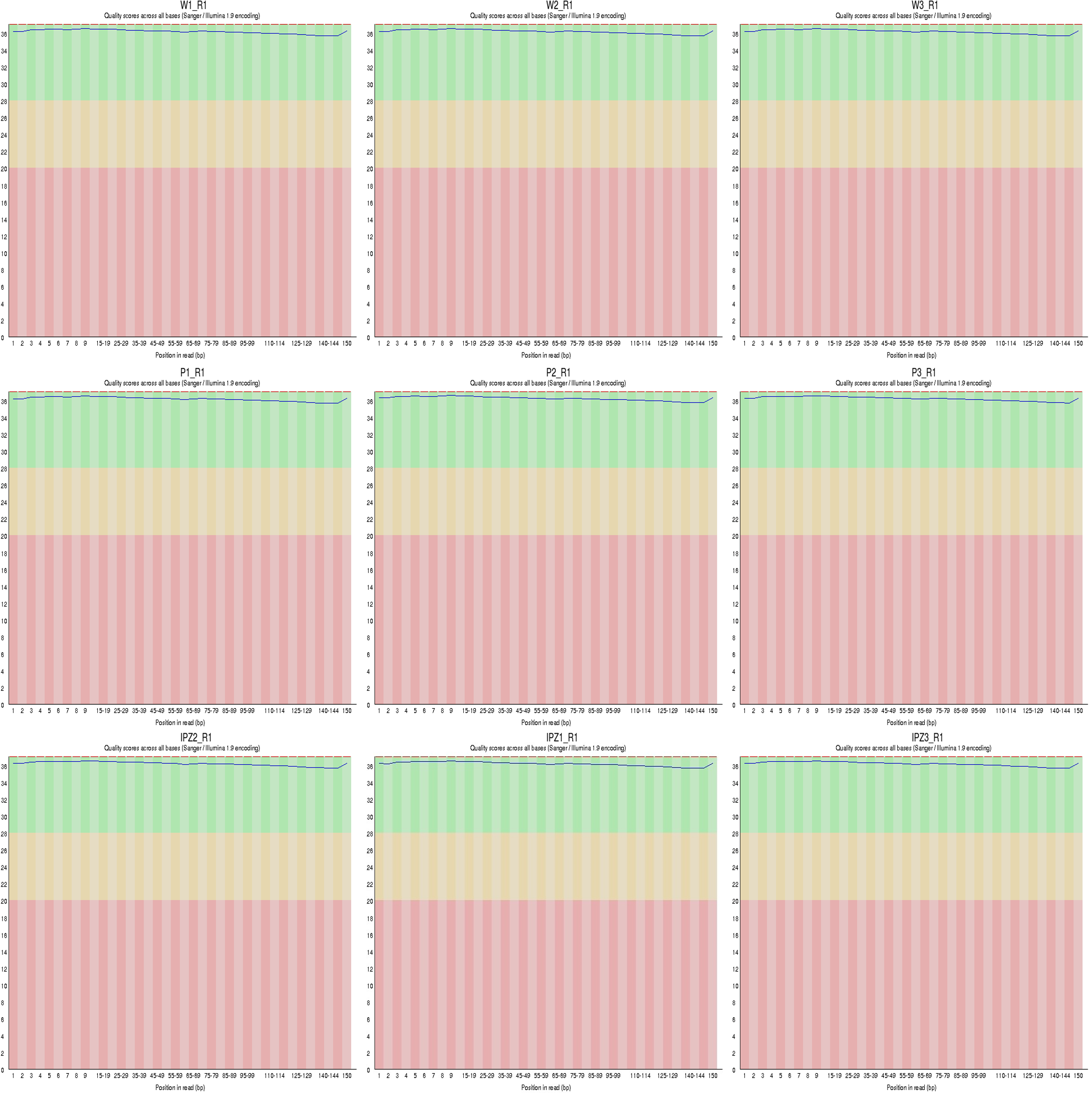

Supplement: S1 Data — (ZIP) [file pgen.1011411.s017.zip › S17 Data/FigS8/WT_R86S_IPZ_QC.png]
