# Supplementary material for: NEMF mutations in mice illustrate how Importin-β specific nuclear transport defects recapitulate neurodegenerative disease hallmarks
Source: PLoS Genet. 2024 Sep 23;20(9):e1011411. doi: 10.1371/journal.pgen.1011411 (PMC11449308; doi:10.1371/journal.pgen.1011411)
Supplement: Attachment — Submitted filename: Sher Response to Reviewers PLoS Genetics.pdf [file pgen.1011411.s018.pdf]

Stony Brook University | The State University of New York

Roger B. Sher, PhD
Assistant Professor
Department of Neurobiology & Behavior & Center for Nervous System Disorders
546 Centers for Molecular Medicine

roger.sher@stonybrook.edu
Office: 631 632 9664
Cell: 207 949 3678

Fax: 631 632 6661

April 1, 2024

Dear PLoS Genetics Editors,

This is to inform you of revisions to the manuscript that was originally submitted to PLoS Biology and is being transferred to PLoS Genetics.

We have updated/edited the abstract to make the significance of our results clearer. We have also added sections such as Author Contributions and Author Summary. We have not added, deleted, or changed any data or analysis or figures. This is entirely based on comments from colleagues here at Stony Brook University who gave us suggestions on how to better direct our submission.

Thank you for your consideration,

Sincerely yours,
