# Supplementary material for: NEMF mutations in mice illustrate how Importin-β specific nuclear transport defects recapitulate neurodegenerative disease hallmarks
Source: PLoS Genet. 2024 Sep 23;20(9):e1011411. doi: 10.1371/journal.pgen.1011411 (PMC11449308; doi:10.1371/journal.pgen.1011411)
Supplement: Attachment — Submitted filename: Plessis-Belair_review.docx [file pgen.1011411.s019.docx]

In the manuscript “Importin-β specific nuclear transport defects recapitulate phenotypic and transcriptional alterations seen in neurodegeneration” from Plessis-Belair et al., the authors describe how mutations to the Nuclear Export Mediator Factor (NEMF) protein elicits errors in nucleocytoplasmic transport (NCT) as well as functional changes in gene expression, including genes that are relevant for neurodegenerative diseases. The authors show very clearly that the NEMF mutation, R86S, causes robust NCT defects in both living animals and in cultured mouse embryonic fibroblasts (MEFs). However, there are a number of significant revisions that need to be performed for this manuscript to be published. First, the mutations investigated in this paper do not appear to be among those identified in patients (e.g., those included in Martin et al., *Nat. Comm.* 2020). The mutations in this model test a loss-of-function hypothesis, but cannot speak to the precise role of human disease-associated mutations. Second, the authors want to relate their findings to neurodegenerative disorders, but the majority of their experiments are performed in non-neuronal cell lines. Thus, the authors should use more careful language when extrapolating their findings to neurodegeneration. Moreover, NEMF has not directly been associated with ALS; the authors should be more cautious with this language as well. Third, there are fundamental questions of ribosomal biology and the role of protein expression and quality control that these studies may be impacted by. The current set of experiments is rather limited in this area and the overall impact of the manuscript would be enhanced by a more detailed examination of the canonical function of NEMF. Fourth, the manuscript in general lacks sufficient background information and detail, leaving the reader without the proper context necessary to appreciate the findings outlined in the paper. Additional specific recommendations are outlined below. Bolded comments are what this reviewer believes to be the most important for the manuscript to be published:

Comments that are not line-specific:

- **Figure legends need to be more detailed. Explain what data are being shown, what data points/error bars/colors represent, etc.**
- Panels within figures are not referenced in order; should be rearranged to appear sequentially within the text
- Replace all eszetts (ß) with betas (β)
- All ums should be changed to µm
- **Each channel for microscopy images needs to be shown separately**
- **As a component of the RQC pathway, it is important to stain for some other marker of nucleosomes (e.g., NPM1) and for ribosomal proteins.**

General questions:

**Does NEMF have an NLS? How does it get into the nucleus itself? Is it Impβ-dependent?**

**Are mice/cells het or hom for NMEF mutants?**

Are MEFs an appropriate cell line to use here? Perhaps good for basic biology questions, but may not be the best for making neuronal conclusions.

**Is protein synthesis affected in NMEF mutant lines due to changes in ribosome QC? Could differences in protein expression be affecting NCT?**

Fig. 1:

A: Should have each channel shown independently. Very difficult to see DAPI and ChAT staining in merged image

F-J: What do the colors represent? N = cells? How many animals? Is each color one animal?

Fig. S1:

The NEMF and merged images are not the same

C and D should have insets for the cells indicated by the arrows; for NEMF in particular it is very difficult to see the phenotype being indicated; insets should be shown for all three channels in addition to the merged

Fig. 2:

A/C: labeling is very confusing; it looks like the rows should be GFP/mCherry, then merge

B/D: what do the colors represent? N = cells? How many animals? Is each color one cell line?

F: Color pattern is different for R86S+NEMF KD

Fig. S2:

Get rid of red squiggle in workflow screenshot

The nuclei in D that were incubated with 70 kDa dextran look much less round; **can the authors comment on this?** Are these nuclei representative? **Also no scale bar is provided – this needs to be added**

A: **KD of NEMF in mutant line has not occurred – this is not mentioned at all in the text, but the graph in the main figure includes it as a category. This is incredibly misleading.** Also, assuming SRC is some sort of scrambled control? Not clear at all. And, **how were these data quantified? Normalized to what housekeeping gene? The methods section is insufficient here.**

Fig. 3:

B: why abundance and not just number?

C: all y-axes should start at zero; also font on axes (B-D) looks distorted

E: font needs to be bigger

Fig. 4:

See questions raised in line 200 regarding microscopy settings

A: the cytoplasmic puncta for Ran are quite striking – has this phenotype been observed in other models?

H: need some loading control for insoluble fraction (perhaps total protein?)

J: what percentage of cells show pTDP+ staining? This figure makes it seem like it is a very high percentage, which is not consistent with previous work on pTDP-43 in disease tissue.

Fig. S4:

C: images need quantification and scale bars. NUP50 is not very convincing from the images shown. Also, mutant cells look unhealthy in general and nuclei look much smaller – is this representative?

Fig. 5:

H,I: why is there no TDP-43 staining for these blots?

Fig. 6:

**J and K have nothing to do with NEMF??? Unclear why these are being shown in the main text figure; they should be shown alongside siNEMF data and images that were collected in the same experiment.**

Main Text:

Line 152: how is disease onset defined? Is this based on the previous work, or was there independent validation of phenotypes (NMJ loss, axonal degeneration)?

**Line 153: haven’t actually proven that this is toxic yet**

Line 164: why does GFP without an NLS get into the nuclei of mutant cells better than the GFP-3X-NLS? Is Impβ binding to the construct and keeping it in the cytoplasm? If it were binding to Impβ, you might expect to see punctate structures in the cytoplasm, which may be there, but it is difficult to determine from one image. **Would be interesting to co-stain for Impβ to see whether this construct is getting trapped, especially given the data shown in Fig. 3.**

Line 178: if pores are leaky, why isn’t the GFP-3X-NLS getting into the nucleus more? Further reason to do the experiment mentioned above, i.e., co-staining for Impβ.

180: need more explanation here about (1) how LTN1 KD relates to RQC, and (2), what these results mean biologically

Line 182: a slight increase relative to what? **Are the WT and R86S columns the same data from 2B? Need to report if so**; and if these were done as separate experiments, it might not be good to compare, due to potential differences in transfection efficiency. **Also, a western blot should be performed to determine whether the GFP construct is expressed at equal levels across cell lines.**

Line 200: **were images taken with the same laser settings? Were images processed using the same settings? Not listed in methods**, and it looks like there are many foci in both cell lines, and intensity can be altered by many factors not related to biology. Cells also have different sizes (shown also in Fig. S4). Smaller cells with the same number of foci would necessarily increase the number of foci per unit area, which may be a confounding factor in interpreting these results. Also, a smaller cell volume could induce crowding; is this a biological effect or a physical one?

Line 202: **you cannot say anything about the strength of the interaction without doing additional experiments.**

Line 205: should cite prior literature on NIR sequestration in disease (reviewed here: <https://academic.oup.com/brain/article/144/10/2915/6279742#316491413>)

Line 218: **not sequestered in “these” puncta – there is no co-staining so these puncta may be completely independent of other puncta shown.**

Line 222: **the level of pTDP staining is highly unusual and does not match previous work or the author’s own image shown in panel A of the same figure. These results may need to be rethought.**

Line 229: **The extent of pTDP-43 staining is not consistent with what others have found in other disease models. The authors should perform additional validation of these results – perhaps a western blot?**

Line 237: does this need a citation?

Line 238: **should look for cryptic exons or other evidence of missplicing events**

Line 242: are these genes related to disease? A specific function? Unclear why they are being singled out over others.

Line 245: need citations for CHMP2B.

Line 248: **the authors should comment on why their MEF model is so divergent from brain; also in line 255, it seems that these observations show that there is transcriptional regulation in MEFs, and maybe in spinal cord, but not in brain.**

Line 267: **why isn’t *Apoe* reduced if this is supposed to be an Importin-β-driven event?**

Line 271: was this sentence supposed to come earlier?

Line 288: **MEF model, not mouse model? Very big difference in conclusion; the mouse *in vivo* data do not seem to show as strong of a phenotype**

Line 309: Fig. S8D? unclear why the authors are switching between cell models so much; the HEK cells do not seem to be adding anything to the story

Methods:

**How are MEFs extracted/isolated?**

For IF staining: **how many cells were counted per experiment?** How were images processed for figures? What laser parameters were used?

qPCR: were all qPCR reactions run with a corresponding control in the same plate?

“Protein Tissue Extraction” is confusing

Minor comments:

Line 97: 97% of what? 40% of what? Cases? Cells?

Line 139: Fig 1?

Line 146: is lamina IX associated with disease phenotype? May be good to cite some literature here (e.g., <https://pubmed.ncbi.nlm.nih.gov/2061715/>)

Line 200: can the antibody on which each PLA probe attaches affect the results? Might it be worth trying to do the reciprocal experiment here?

Line 215: co-stain for some proteins? It would be interesting to see if these aggregates are all the same structures

Line 407: were should be was

Replace “deg” with degree symbol, subscript numbers in chemical formulae (CO_2_, MgCl_2_)
