# Supplementary material for: NEMF mutations in mice illustrate how Importin-β specific nuclear transport defects recapitulate neurodegenerative disease hallmarks
Source: PLoS Genet. 2024 Sep 23;20(9):e1011411. doi: 10.1371/journal.pgen.1011411 (PMC11449308; doi:10.1371/journal.pgen.1011411)
Supplement: Attachment — Submitted filename: Plessis-Belair_Sher_Reviewer_Responses.docx [file pgen.1011411.s020.docx]

**Reviewer Comments**

In the manuscript “Importin-β specific nuclear transport defects recapitulate phenotypic and transcriptional alterations seen in neurodegeneration” from Plessis-Belair et al., the authors describe how mutations to the Nuclear Export Mediator Factor (NEMF) protein elicits errors in nucleocytoplasmic transport (NCT) as well as functional changes in gene expression, including genes that are relevant for neurodegenerative diseases. The authors show very clearly that the NEMF mutation, R86S, causes robust NCT defects in both living animals and in cultured mouse embryonic fibroblasts (MEFs). However, there are a number of significant revisions that need to be performed for this manuscript to be published.

First, the mutations investigated in this paper do not appear to be among those identified in patients (e.g., those included in Martin et al., *Nat. Comm.* 2020). The mutations in this model test a loss-of-function hypothesis, but cannot speak to the precise role of human disease-associated mutations.

We thank the reviewer for this request for clarification. The two mouse mutations were ENU induced point mutations that led to two independent missense variants. Martin 2020 showed that there is no loss of NEMF protein in either of these mutants. The human patient mutations are variable, with homozygous nonsense mutations ad frameshift mutations (pArg870*, p.Lys672*, pSer817fs*7, p.Asp959fs*2, pArg671*) along with one patient with two maternal missense (pArg327Gln+p.Pro926Leu combined with a paternal frameshift p.Lys923Argfs*27), and one patient with heterozygous missense (p.Ile553Thr/+). We have not recapitulated any of these patient mutations at this time in mouse or human cells, but have relied on the available mouse lines where NEMF was initially identified as being related to a neurological defect. The range of onset for the patients is from 6 months to 28 years, showing that NEMF mutations results in a range of phenotypes in human patients just as we have seen in our two ENU-mouse models. None of the clinicians who worked with these patients were able to get permission to collect any patient tissue, except for one where the frozen tissues are no longer viable coming out of storage. Because many of the human mutations are nonsense homozygous, the assumption is that there is a loss of function of NEMF.

Second, the authors want to relate their findings to neurodegenerative disorders, but the majority of their experiments are performed in non-neuronal cell lines. Thus, the authors should use more careful language when extrapolating their findings to neurodegeneration. Moreover, NEMF has not directly been associated with ALS; the authors should be more cautious with this language as well.

We agree that many of the experiments were performed in non-neuronal cell lines, but we have included significant data in the revision on neuronal lines that recapitulate the results found in our MEFs. In addition, the biochemical pathways known to be affected in neurodegenerative diseases in human neuronal tissues are also found in our MEFs, indicating their usefulness for the basic understanding of how NEMF functions in these pathways.

Third, there are fundamental questions of ribosomal biology and the role of protein expression and quality control that these studies may be impacted by. The current set of experiments is rather limited in this area and the overall impact of the manuscript would be enhanced by a more detailed examination of the canonical function of NEMF.

We agree that the role of NEMF in ribosomal biology is an important consideration, and we have therefore included substantial data in the revision looking at translational processes and the independence of the nuclear import defect from ribosomal quality control function.

Fourth, the manuscript in general lacks sufficient background information and detail, leaving the reader without the proper context necessary to appreciate the findings outlined in the paper.

We agree that the initial submission was lacking in these areas, and we have expanded our explanations and background context in both the introduction and discussion to address this.

Additional specific recommendations are outlined below. Bolded comments are what this reviewer believes to be the most important for the manuscript to be published:

Comments that are not line-specific:

- **Figure legends need to be more detailed. Explain what data are being shown, what data points/error bars/colors represent, etc.**
  - Thank you for your attention to this, we updated our figure legends to be more specific on what is being shown in each figure.
- Panels within figures are not referenced in order; should be rearranged to appear sequentially within the text
  - We adjusted the panels within figures to be referenced more linearly.
- Replace all eszetts (ß) with betas (β)
  - Thank you for catching this, this was a mistake as a result of working on mac/windows computers.
- All ums should be changed to µm
  - Thank you for catching this, units have been adjusted accordingly.
- **Each channel for microscopy images needs to be shown separately**
  - We separated the channels for all target proteins for better visualization
- **As a component of the RQC pathway, it is important to stain for some other marker of nucleosomes (e.g., NPM1) and for ribosomal proteins.**
  - We have included an NEMF and NPM1 co-stain as well as ribosomal stains.

General questions:

**Does NEMF have an NLS? How does it get into the nucleus itself? Is it Impβ-dependent?**

**Are mice/cells het or hom for NMEF mutants?**

NEMF has not been described to contain an NLS. It was initially described in flies to interact with a Nuclear Export Signal (Bi et al 2005), but since then most of its investigations are on its role in the RQC. These mice and MEFs are homozygous for NEMF mutants. We have analyzed the protein sequence for human and mouse NEMF through Novopro labs NLS predictor (based on Nguyen Ba AN, Pogoutse A, Provart N, Moses AM. NLStradamus: a simple Hidden Markov Model for nuclear localization signal prediction. BMC Bioinformatics. 2009;10:202.) and have found a very strong putative NLS in the C-terminal region (KGKKGKKGK(T/P)KDEPVKK(Q/P)), which has a probability of 0.960 (human) and 0.990 (mouse) for being an Importin-B style classical NLS. Further work will clarify the putative NLS function of this sequence.

Are MEFs an appropriate cell line to use here? Perhaps good for basic biology questions, but may not be the best for making neuronal conclusions.

As you highlight here, the use of MEFs allows us to ask basic biology questions on the molecular functions of the R86S mutant and downstream pathways. This, with the inclusion of our *in vivo* studies looking at spinal cord motor neurons as well as the addition of primary neurons, allows us to make connections between the pathologies we see *in vitro* and compare them to what we observe *in vivo*. The ability to make both make direct connections, as well as compare differences between the cell types allows us to make better inferences on the aberrant pathways.

**Is protein synthesis affected in NEMF mutant lines due to changes in ribosome QC? Could differences in protein expression be affecting NCT?**

RQC is often considered to not affect translation and purely be a post-translation quality control mechanism. However, changes in growth rate/cell size do highlight that there could be protein synthesis issues at play. Therefore, we included a global synthesis puromycin assay to investigate this very question.

Fig. 1:

A: Should have each channel shown independently. Very difficult to see DAPI and ChAT staining in merged image

As indicated above, we have separated the channels for all target proteins for better visualization.

F-J: What do the colors represent? N = cells? How many animals? Is each color one animal?

As indicated above, we have adjusted the figure legends to better explain this in the text.

Fig. S1:

The NEMF and merged images are not the same

Thank you for catching this mistake.

C and D should have insets for the cells indicated by the arrows; for NEMF in particular it is very difficult to see the phenotype being indicated; insets should be shown for all three channels in addition to the merged

We have adjusted these images to separate all channels and added

Fig. 2:

A/C: labeling is very confusing; it looks like the rows should be GFP/mCherry, then merge

Thank you for catching this mistake, we adjusted the figure.

B/D: what do the colors represent? N = cells? How many animals? Is each color one cell line?

F: Color pattern is different for R86S+NEMF KD

Thank you for catching this mistake, we adjusted the figure and figure legend.

Fig. S2:

Get rid of red squiggle in workflow screenshot

The nuclei in D that were incubated with 70 kDa dextran look much less round; **can the authors comment on this?** Are these nuclei representative? **Also no scale bar is provided – this needs to be added**

Reviewer # 2 had made a similar comment on the dextran exclusion assay and suggested that we switch to a permeabilized assay instead of nuclear isolation. With these changes, we hope to answer these concerns.

A: **KD of NEMF in mutant line has not occurred – this is not mentioned at all in the text, but the graph in the main figure includes it as a category. This is incredibly misleading.** Also, assuming SRC is some sort of scrambled control? Not clear at all. And, **how were these data quantified? Normalized to what housekeeping gene? The methods section is insufficient here.**

KD of NEMF in the mutant unexpectedly resulted in a large amount of cell death, resulting in a lower knockdown of the mutant *Nemf* (~20%) vs WT *Nemf* (~%54%). We therefore adjusted the text and figures to demonstrate this discrepancy without drawing major conclusions from the lack of a knockdown, including removing those data points as necessary. We agree that proceeding with transparency in the knockdown as indicated in the results is best.

Fig. 3: now Fig 4

B: why abundance and not just number?

We adjusted this figure to report only number.

C: all y-axes should start at zero; also font on axes (B-D) looks distorted

The figures here were adjusted to begin at 0.

E: font needs to be bigger

The font size has been adjusted.

Fig. 4: now Fig 5

See questions raised in line 200 regarding microscopy settings

A: the cytoplasmic puncta for Ran are quite striking – has this phenotype been observed in other models?

Thank you for this concern, we have replaced this image with a co-stain with Importin-B and which will hopefully help to shed light on this matter.

H: need some loading control for insoluble fraction (perhaps total protein?)

Thank you for this suggestion, we have included a stain-free total protein control with this figure.

J: what percentage of cells show pTDP+ staining? This figure makes it seem like it is a very high percentage, which is not consistent with previous work on pTDP-43 in disease tissue.

Thank you for this concern, following comments from reviewer #2, these samples were re-stained adjusting antibody concentrations to reduce background. From these new images, we calculated the percentage of cells showing pTDP43 inclusions to represent the population observed.

Fig. S4: now Fig S5

C: images need quantification and scale bars. NUP50 is not very convincing from the images shown. Also, mutant cells look unhealthy in general and nuclei look much smaller – is this representative?

Thank you for this concern. We adjusted these images to show Nup153, Nup98, as well as NPC antibody mAb414 and co-stained with Importin-B to show that these Nups colocalize to similar structures as seen with NTRs. The population observed is representative of the cell line.

Fig. 5: now Fig 6

H,I: why is there no TDP-43 staining for these blots?

Thank you for catching this, we have removed the TDP-43 stain from G.

Fig. 6: now Fig 7

**J and K have nothing to do with NEMF??? Unclear why these are being shown in the main text figure; they should be shown alongside siNEMF data and images that were collected in the same experiment.**

Thank you for this comment, we have included Importin-beta upregulation experiments, and we have relocated the IPZ-treated human neuronal cell line work shown in figure J and K to the supplementary figures.

Main Text:

Line 152: how is disease onset defined? Is this based on the previous work, or was there independent validation of phenotypes (NMJ loss, axonal degeneration)?

Thank you for this comment. The work that validated the phenotypes occurred in parallel to the work shown here with Dr. Sher being part of the investigative team from the Martin et al 2020 paper describing NMJ loss and axonal degeneration. Therefore, there was no independent validation of these phenotypes. Although, we have observed similar onset of pathology and time of death in our mouse colonies.

**Line 153: haven’t actually proven that this is toxic yet**

This line has been adjusted.

Line 164: why does GFP without an NLS get into the nuclei of mutant cells better than the GFP-3X-NLS? Is Impβ binding to the construct and keeping it in the cytoplasm? If it were binding to Impβ, you might expect to see punctate structures in the cytoplasm, which may be there, but it is difficult to determine from one image. **Would be interesting to co-stain for Impβ to see whether this construct is getting trapped, especially given the data shown in Fig. 3.**

Thank you for this suggestion. We agree that co-stains would better indicate the events occurring in this figure. Therefore, we included co-stains for images in Figure 2, which were included in Fig S2D-E. We do observe some colocalization of these structures in the cytoplasm, but this co-localization is diffuse. The lack of puncta could be a result of the diffuse nature of the GFP-3X-NLS substrate, but it is difficult to speculate.

Line 178: if pores are leaky, why isn’t the GFP-3X-NLS getting into the nucleus more? Further reason to do the experiment mentioned above, i.e., co-staining for Impβ.

We agree that there may be some interaction with Importin-beta in the cytoplasm. Therefore, we have included the co-stains indicated above. However,

180: need more explanation here about (1) how LTN1 KD relates to RQC, and (2), what these results mean biologically

Thank you for highlighting this to us, this was adjusted in the text.

Line 182: a slight increase relative to what? **Are the WT and R86S columns the same data from 2B? Need to report if so**; and if these were done as separate experiments, it might not be good to compare, due to potential differences in transfection efficiency. **Also, a western blot should be performed to determine whether the GFP construct is expressed at equal levels across cell lines.**

Thank you for catching this. The experiments shown in Figure 2 were run in parallel but were separated for visualization. We have adjusted the text and the figure to statistically compare these assays. We have also included a western of the GFP and GFP-3X-NLS constructs in Fig S2.

Line 200: **were images taken with the same laser settings? Were images processed using the same settings? Not listed in methods**, and it looks like there are many foci in both cell lines, and intensity can be altered by many factors not related to biology. Cells also have different sizes (shown also in Fig. S4). Smaller cells with the same number of foci would necessarily increase the number of foci per unit area, which may be a confounding factor in interpreting these results. Also, a smaller cell volume could induce crowding; is this a biological effect or a physical one?

Thank you for this clarification. The images were taken with the same settings for both control and treatment groups. This was better reported in the methods section. We adjusted Fig3, now Fig4 to represent the PLA foci per cell instead of per area to remove possible crowding confounds.

Line 202: **you cannot say anything about the strength of the interaction without doing additional experiments.**

Thank you for highlighting this. We adjusted this in the manuscript.

Line 205: should cite prior literature on NIR sequestration in disease (reviewed here: <https://academic.oup.com/brain/article/144/10/2915/6279742#316491413>)

Thank you for this suggestion, this was included in the text.

Line 218: **not sequestered in “these” puncta – there is no co-staining so these puncta may be completely independent of other puncta shown.**

Thank you for highlighting this. To account for this, we replaced the images with co-stains, co-staining the proteins of interest with Importin-beta.

Line 222: **the level of pTDP staining is highly unusual and does not match previous work or the author’s own image shown in panel A of the same figure. These results may need to be rethought.**

As previously stated, these samples were re-stained adjusting antibody concentrations to reduce background. From these new images, we calculated the percentage of cells showing pTDP43 inclusions.

Line 229: **The extent of pTDP-43 staining is not consistent with what others have found in other disease models. The authors should perform additional validation of these results – perhaps a western blot?**

Thank you for this suggestion. Our intentions were to also perform a western blot for pTDP-43, but unfortunately, the pTDP-43 antibody was unsuccessful on a western. To compensate for this, we performed the sarkosyl insoluble fractionation to isolate insoluble forms of TDP-43.

Line 237: does this need a citation?

Thank you for this suggestion, we included a citation for TDP-43 transcriptional dysregulation.

Line 238: **should look for cryptic exons or other evidence of missplicing events**

Thank you for this suggestion, we have included bonafide TDP-43 mouse targets such as Sort1 and Dnjac5 in FigS7 to show mis-splicing events in this mutant model as previously described in Polymenidou et al, *Nat Neurosci.* 2011.

Line 242: are these genes related to disease? A specific function? Unclear why they are being singled out over others.

These genes were highlighting due to their disease relevance in neurodegeneration, where these genes are commonly observed to be dysregulated. To highlight this, citations were included.

Line 245: need citations for CHMP2B.

As stated above, citations for CHMP2B were included due highlight disease relevance.

Line 248: **the authors should comment on why their MEF model is so divergent from brain; also in line 255, it seems that these observations show that there is transcriptional regulation in MEFs, and maybe in spinal cord, but not in brain.**

This is mostly due to the bulk extraction of the tissue and/or onset of pathology.

Line 267: **why isn’t *Apoe* reduced if this is supposed to be an Importin-β-driven event?**

We believe that Apoe induction may have both Importin-beta dependent and independent regulation, or potentially that it has a differential sensitivity or timeframe of alteration to Importin-B changes than other gene products.

Line 271: was this sentence supposed to come earlier?

Thank you for catching this, this line was adjusted.

Line 288: **MEF model, not mouse model? Very big difference in conclusion; the mouse *in vivo* data do not seem to show as strong of a phenotype**

Thank you for highlighting this, we are comparing cell lines here and therefore have adjusted the text to reflect this.

Line 309: Fig. S8D? unclear why the authors are switching between cell models so much; the HEK cells do not seem to be adding anything to the story

We believe that the inclusion of HEK cells allows us to connect our findings in mouse fibroblast cell lines to human fibroblast cell lines.

**How are MEFs extracted/isolated?**

A description of this isolation process was included in the methods section.

For IF staining: **how many cells were counted per experiment?** How were images processed for figures? What laser parameters were used?

This varies per experiment and was therefore included in the individual figure legends. Images processing and laser parameters were included in the methods section.

qPCR: were all qPCR reactions run with a corresponding control in the same plate?

Yes all corresponding controls were run on the same plate. This was included in the methods section.

“Protein Tissue Extraction” is confusing

We apologize for the confusion, we have adjusted the text.

Minor comments:

Line 97: 97% of what? 40% of what? Cases? Cells?

Thank you for catching this, this was adjusted in the text.

Line 139: Fig 1?

Thank you for catching this error, this was adjusted in the text.

Line 146: is lamina IX associated with disease phenotype? May be good to cite some literature here (e.g., <https://pubmed.ncbi.nlm.nih.gov/2061715/>)

Thank you for the suggestion, this was included within the text.

Line 200: can the antibody on which each PLA probe attaches affect the results? Might it be worth trying to do the reciprocal experiment here?

If the reviewer is suggesting that in the original PLA experiment, we used a rabbit NEMF primary and a mouse IgG2a Importin Beta primary, and therefore we should try to use a mouse IgG2a NEMF primary with a rabbit Importin Beta primary, the problem is that there is no good non-rabbit NEMF antibody available. We have tried multiple antibodies for NEMF and have only found the rabbit one to give consistent results.

Line 215: co-stain for some proteins? It would be interesting to see if these aggregates are all the same structures

This was included in from the earlier suggestion.

Line 407: were should be was

Replace “deg” with degree symbol, subscript numbers in chemical formulae (CO_2_, MgCl_2_)

Thank you for catching this, this was adjusted in the text.

Reviewer #2: Altered nucleocytoplasmic transport (NCT) capacity has been implicated in multiple neurodegenerative diseases. However, it remains unclear as to whether this phenomenon is a driver of toxicity in disease. In this study, Plessis-Belair and colleagues identify and characterize NCT alterations and hallmarks of neurodegenerative pathology in a mouse model based on the expression of mutant NEMF, a gene previously linked to neurodevelopmental alterations. The authors demonstrate that this model shares many features of neurodegenerative disease including impaired nuclear import, TDP-43 pathology, and transcriptional alterations. Additionally, the authors provide evidence that transient inhibition of importin B alone is sufficient to trigger disease associated pathology. While of potential interest and significance to a broad readership, there are a number of improvements that should be made to strengthen the overall conclusions and completeness of the study prior to publication. These are detailed below.

1. The study and/or manuscript would benefit from a better description of the mouse model used. For example, is mutant NEMF expressed throughout development? Is this a conditional model? Is mutant NEMF expressed in all cell types? Is mutant NEMF OE or is this a CRISPR generated mutation? Can the authors please detail additional hallmarks of neurodegeneration such as neuronal loss, GFAP immunoreactivity, Iba1 immunoreactivity, survival deficits, behavioral abnormalities etc?

Thank you for stressing this in our background information. The mutant NEMF is expressed throughout development. The R86S and R487G models are derived from 2 ENU derived mutations and therefore are consistently expressed. In the recent paper in which Dr. Roger Sher was part of the investigative group, the mice were described to have loss of NMJs, hindlimb wasting, and axonal degeneration. The R86S mice had an early onset with a median lifespan of 20 days, whereas the R487G mice were much more long-lived, with many living passed 1 year, but showed symptoms as early as 8 weeks. We have adjusted the background to include more details on these mouse models.

2. Are the phenotypes observed in MEFs recapitulated in CNS cell types (ie primary neuron or glial cultures from the mutant mice)?

Thank you for this suggestion, we have isolated primary neuronal cells from control and mutant mice to demonstrate that some of these transcriptional alterations also exist in these cell types.

3. Cell based studies appear to rely on OE of mutant NEMF. Could the authors provide evidence that endogenous levels of mutant NEMF induce similar pathophysiological phenomena?

Thank you for highlighting this clarification, the MEFs are isolated from ENU-induced homozygous mutant R86S mice and therefore are not overexpressing NEMF. We have adjusted the text to better clarify this.

4. The authors state that Nups and NTRs are “sequestered” in cytoplasmic puncta in the context of mutant NEMF expression. However, there is little evidence to support this. In particular, the typical expression patterns of the proteins evaluated appear to remain largely intact. Thus, it is unclear if this is truly sequestration away from normal function or simply a few molecules trapped in abnormal cytoplasmic puncta. What percentage of molecules are in puncta as opposed to normal function and is this sufficient to recapitulate inhibition or loss?

Thank you for this suggestion, we have updated the images of Nups with co-stains with Importin-beta, where we can observe the colocalization of these reporters in the cytoplasm. Furthermore, we have quantified the percentage of nuclear Nups and found a reduction in nuclear signal for the Nups highlighted in this manuscript.

5. Does OE or activation of importin B reverse or prevent pathologic alterations in mutant NEMF model?

Thank you for this suggestion along with reviewer #3. We have included Importin-B overexpression assays in Fig7 J-M to highlight that overexpression of Importin-B is capable of reversing/preventing some of this pathological alterations.

6. Although NCTs reporters suggest that transportin mediated nuclear import and nuclear export are not disrupted, can the authors please perform immunostaining to determine if their localization is intact or altered?

Thank you for this suggestion, we have included images of transportin-1 and exportin-1 in FigS5A to highlight that their localization is intact in the mutant cells.

7. For the dextran assay, it is unclear why the authors decided to isolate nuclei as opposed to using more conventional permeabilized cell assays. Importantly, the nuclei look rather disrupted and dextran molecules in and outside of the nucleus are hard to see given the lack of individual channel images presented.

Thank you for this suggestion, as reported in FigS2G-H, we utilized a permeabilized cell dextran exclusion assay to report our findings. We were able to demonstrate similar results as our isolated nuclei, but hope this clarifies any doubts.

8. Are mutant NEMF cells dying in culture? Many images presented appear to contain a number of unhealthy looking cells.

The cell line has been immortalized, highlighting that these cells are actively dividing. Despite this, the cells are vulnerable to outside stressors. Prior stains and observations into caspase-3 activity has not highlighted any overt cell death markers in these cells without the addition of a stressor.

9. IPZ and IVM are clearly toxic to cells. Can the authors please comment on whether transcriptomic changes are truly a reflection of the effects of impaired NCT or simply a reflection of cell death cascades?

Prior to these experiments, we optimized the level of IPZ or IVM required to recapitulate a nuclear import block without causing over toxic effects. This is highlighted in Fig S9A,B where concentrations chosen for these experiments did not result in any significant cell death after 48 hours. Cell death cascades were also not highlighted in our RNA seq data.

10. Can the authors please demonstrate that their importazole treatment blocks import (importin B vs transportin) as expected via use of NCT reporters?

Thank you for this suggestion, we included this in Fig S2 with the use of the GFP-3X-NLS and PY-NLS reporters.

11. siNEMF recapitulates transcriptional alterations but earlier experiments presented suggest that NEMF KD does not recapitulate mutant NEMF phenotypes. These seemingly discrepant results would benefit from additional discussion.

Thank you for this comment. We believe that siNEMF treatment and mutant NEMF cells have different degrees of pathology, but the recapitulation of transcriptional alterations suggests that the pathways might be Importin-Beta dependent. The presence of cytoplasmic puncta in the siNEMF treated MEFs suggests some aberrant interaction with the 3X-NLS reporter, possibly directly through Importin-Beta or through some form of aggregation induced sequestration as suggested by the presence of cytoplasmic puncta.

12. NEMF and Importin B staining and localization appear quite different in MEFs (Fig 3) vs in vivo (Fig 1). Staining in motor cortex (S1) vs spinal cord (Fig 1) also appear quite different even in WT mice. Can the authors comment on this and/or the specificity of antibodies used?

The same NEMF antibody was used for all assays as indicated in the supplementary tables. There are differences in the brains vs the spinal cord as well as in MEFs in terms of the nuclear localization of NEMF. For Spinal cord, we observe a diffuse nuclear signal concentrated at the nucleolus. For Brain and MEFs, we observe more of a concentrated nucleolar signal, but also have observed diffuse staining in the MEFs.

13. PLA results are difficult to interpret as presented. It appears the majority of the NEMF signal is within the nucleus and importin B granules are largely not colocalizing with cytoplasmic NEMF. Yet there are abundant PLA signals in the cytoplasm. Can the authors please comment on antibody specificity and include higher magnification images of no primary and no probe controls for PLA to ensure specificity of all signals?

The antibodies used in Fig 4A are the same antibodies reported for Fig4F. Higher magnification images were included in the supplemental which show little to no off target signals.

14. In S5, much of the pTDP signal appears to fill the nucleus in an age dependent manner. This is not typical of pTDP immunoreactivity in other neurodegenerative mouse models where signal is observed as cytoplasmic puncta. Can the authors please comment on this and/or verify their findings with conventional DAB based IHC?

Thank you for addressing this. We re-stained these tissues adjusting antibody concentrations. The staining should better reflect typical pTDP43 immunoreactivity.

15. TDP-43 regulatory sequences are not conserved in mouse vs human STMN2 RNA. Thus, it is well known that STMN2 does not represent a TDP-43 target in mouse models. As a result, it is unclear whether downregulation of STMN2 in NEMF MEFs actually reflects TDP-43 loss of function pathology. It is suggested that the authors evaluate bonafide mouse TDP-43 targets.

We agree that STMN2 TDP43-dependent regulatory sequences are not conserved in mouse, and therefore the fact that we recapitulate STMN2 downregulation in our mouse model is an indication that TDP43-dependent control is only a part of the regulation of STMN2, and that Importin-beta interruption may be another part of that regulation, independent of TDP43. We have included bona fide mouse TDP43 targets in the revised manuscript and have found them to show alterations in splicing, as predicted from TDP43 dysfunction.

16. Could the authors please speculate on the mechanisms by which mutant NEMF or WT NEMF may contribute to altered NCT and neurodegeneration in the discussion?

From our results, we believe that normally, NEMF has some interaction with Importin-Beta, as indicated from the PLA results where WT NEMF still shows some interactions. We believe the mutant NEMF may aberrantly interact with Importin-Beta (PLA results), either directly or indirectly, sequestering Importin-B and not allowing it to properly transport proteins into the nucleus. The NEMF KD experiments, which show similar Importin-B dependent import defects, would support this as loss of NEMF may also impair normal Importin-B function, although not through sequestration, but rather through loss of some (presently unknown) normal NEMF function/interaction.

17. Overall, the title and abstract are written in a manner that suggests the focus of this manuscript is on defining the role of importin B in neurogenerative disease. However, the data and manuscript are largely focused on characterizing NCT defects in an NEMF mouse model. Thus, it is suggested that the title and abstract be re-written to more accurately represent the data presented in this manuscript.

Thank you for this suggestion, we have re-written the abstract and the title of the manuscript to better represent our findings in the paper.

Reviewer #3: In this paper, Plessis-Belair et al. collect evidence that Importin-beta transport defects elicit phenotypic and transcriptional alterations connected with TDP-43 proteinopathies. This study is high quality and quite compelling. Overall, it helps build a narrative that Importin-beta nuclear import dysfunction is likely to be a pathological contributor to disease. I recommend publication once a couple of key issues are addressed:

1. It remains possible that the Importin-β transport defects are a consequence rather than a cause of disease in the NEMF mouse model. Hence It is important to demonstrate that correcting the Importin-beta nuclear import dysfunction could be therapeutic. Thus, can the deficits observed in Figure 4 be corrected by upregulation of importin-beta activity, for example, by overexpressing importin-beta, importin-alpha, or perhaps both as has been suggested by Rossoll and colleagues (PMID: 36482422 & PMID: 38254150).

Thank you for this suggestion. To address this question, we upregulated importin-beta in our MEFs and found the result quite compelling and therefore we included them in the manuscript.

2. By the same logic, can the deficits observed in Figure 4 be exacerbated by importazole.

Again, thank you for this suggestion. IPZ has a great effect on these mutant cells, therefore we performed a viability assay to indicate the toxic effects of IPZ.

Addition of these studies will help confirm or refute the central hypothesis of this paper.
